# Supplementary material for: Identifying malaria risks amongst forest going populations in Mondulkiri province and Kampong Speu province, Cambodia: a large cross-sectional survey
Source: Malar J. 2025 Feb 22;24:59. doi: 10.1186/s12936-025-05290-0 (PMC11847376; doi:10.1186/s12936-025-05290-0)
Supplement: Supplementary file 1 — Supplementary Material 1 [file 12936_2025_5290_MOESM1_ESM.pdf]

01_CrossSectional_VILLAGE_T0

1. ចុចជីភីអេស Collect the GPS coordinates of this village

latitude (x.y °)

longitude (x.y °)

altitude (m)

accuracy (m)

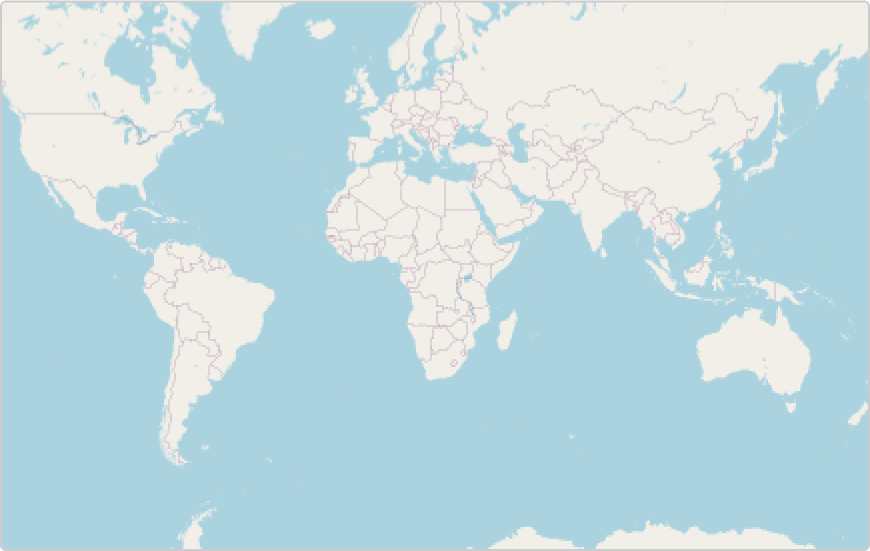

2. កាលបរិច្ឆេទ Enter a date

yyyy-mm-dd

3. លេខមេក្រុម Supervisor Number

4. ខេត្ត Province

- ◯ មណ្ឌលគិរី Mondulkiri
- ◯ កំពង់ស្ពឺ Kampong Speu

5. Mondulkiri Village Name

- 01 ពូត្រំ Tu Trom
- 02 ទួលល្វា Tuol Lvea
- 03 ដេអេ D.A
- 04 ពូជ្រៃចុងផាង Pu Chrei Choung Phang (baro kong)
- 05 អណ្តូងក្រឡឹង Andoung Kraloeng
- 06 ពូចារ Pu Char
- 07 ពូញ៉ាវ Pu Nhav
- 08 តាំងលាំង Tang Lang
- 09 ក្រុមអភិរក្សព្រៃ Ranger

6. Kampong Speu OD Name

- Phnom Sruoch
- Kampong Speu

7. Phnom Sruoch OD Village Name

- Banteay Roka
- Banteay Roka_Kirisenchey (M)
- Dak Por_Toap Mreak (M)
- Doung_Kraong Meanchey (M)
- Krang Chek
- Krasang Khpos
- Peam Lvea
- Peam Lvea_Sre Doung (M)
- Prey Kahiech
- Rumduol Thmei
- Trapeang Chey_Prey Toteoung (M)

8. Kampong Speu OD Village Name

- ○ Anlong Sangkae
- ○ Choam
- ○ Kampeh
- ○ Knong Ay
- ○ Kriel Pong
- ○ Ou Anchar (M)
- ○ Roung Masin
- ○ Ta Sal

9. ឈ្មោះប្រធានភូមិ Village head name

____

10. តើភូមិនេះមានចំនួនខ្នងផ្ទះប៉ុន្មាន? How many households are in this village?

____

11. តើភូមិនេះមានចំនួនប្រជាជនប៉ុន្មាននាក់? What is the total population of the village?

____

12. តើភូមិនេះមានចំនួនប្រជាជនជាភេទប្រុសប៉ុន្មាននាក់? How many males are there?

____

13. តើភូមិនេះមានចំនួនប្រជាជនក្រោម១៨ឆ្នាំប៉ុន្មាននាក់? How many individuals are under 18 years?

____

14. តើភូមិនេះមានចំនួនប្រជាជនក្រោម៥ឆ្នាំប៉ុន្មាននាក់? How many individuals are under 5 years?

____

15. តើភូមិនេះអាចធ្វើដំណើរបានដោយរថយន្ត (ឡាន)ដែរឬទេនៅរដូវវស្សា? Is the village accessible by car during rainy season?

- ○ បាទ/ចាស Yes
- ○ ទេ No

16. តើភូមិនេះអាចធ្វើដំណើរបានដោយម៉ូតូដែរឬទេនៅរដូវវស្សា? Is the village accessible by moto during rainy season?

- ○ បាទ/ចាស Yes
- ○ ទេ No

17.តើភូមិនេះមានភូមិឧបសម្ព័ន្ធដែរឬទេ? Are there any khrom in this village?

- ◯ បាទ/ចាស Yes
- ◯ ទេ No

18. ប្រសិនបើ បាទ/ចាស តើមានប៉ុន្មាន? If yes, How many khrom are there in this village?

____
