# Supplementary material for: Identifying malaria risks amongst forest going populations in Mondulkiri province and Kampong Speu province, Cambodia: a large cross-sectional survey
Source: Malar J. 2025 Feb 22;24:59. doi: 10.1186/s12936-025-05290-0 (PMC11847376; doi:10.1186/s12936-025-05290-0)
Supplement: Supplementary file 2 — Supplementary Material 2 [file 12936_2025_5290_MOESM2_ESM.pdf]

03_CrossSectional_INDIVIDUAL_T0

អ្នកប្រមូលទិន្នន័យ៖ សូមពន្យល់ថាអ្នកនឹងសួរសំណួរមួយចំនួនអំពីគ្រួសារ ដែលពួកគេកំពុងរស់នៅឥឡូវនេះ - រួមទាំងភ្ញៀវដែលមកលេងម្តងម្កាលនៅក្នុងផ្ទះរបស់អ្នកកាលពីយប់មិញ។ Data collector: Please explain you are going to ask some questions about the individual, their household, their travel patterns, and their use of products to prevent mosquito bites. This information will help guide the study for which they have signed up to. We appreciate their answers.

លក្ខខណ្ឌចូលរួម និងទំរង់យល់ព្រម Eligibility and Informed Consent

1. តើអ្នកបុគ្គលហ្នឹងអាចចូលរួមក្នុងការសិក្សាតាមលក្ខខណ្ឌដែរឬទេ? Does this individual meet the inclusion criteria for the study?

- ◯ បាទ/ចាស Yes
- ◯ ទេ No (ឈប់ STOP)

2. តើបុគ្គលហ្នឹងបានអាន ហើយយល់ពីទម្រង់យល់ព្រម និងយល់ព្រមចូលរួមក្នុងការសិក្សានេះដែរឬទេ? Has this individual read and understood the informed consent form, and agree to participate in the study?

- ◯ បាទ/ចាស Yes
- ◯ ទេ No (STOP)

ការប្រមូលសំណាកឈាម និងការធ្វើតេស្តគ្រុនចាញ់ដោយតេស្តរហ័សBlood Collection and Rapid Test

3. តើបានយកសំណាកឈាមពីបុគ្គលនេះដែរឬទេ? Were blood spots collected from this participant?

- ◯ បាទ/ចាស Yes
- ◯ ទេ No

4. ប្រសិនបើបានយកសំណាកឈាម ថាតើបានយកសំណាកឈាមចំនួនប៉ុន្មាន? If blood spots were collected, how many?

____

5. តើបុគ្គលនេះបានធ្វើតេស្តរហ័សរកជម្ងឺគ្រុនចាញ់ដែរឬទេ? Was a malaria rapid test given to this participant?

- ◯ បាទ/ចាស Yes
- ◯ ទេ No

6. ប្រសិនបើបានធ្វើតេស្តរហ័ស តើលទ្ធផលតេស្តយ៉ាងដូចម្តេចដែរ? If a rapid test was given, what was the result?

- ◯ អវិជ្ជមាន Negative
- ◯ វិជ្ជមាន Positive
- ◯ មិនអាចសន្និដ្ឋានបាន Inconclusive

ពត៌មានការប្រមូលទិន្នន័យ Data Collection Information

7. លេខមេក្រុម Supervisor Number

---

8. សូមកត់ត្រាទីតាំងបច្ចុប្បន្ន Record your current location

---

latitude (x.y °)

longitude (x.y °)

altitude (m)

accuracy (m)

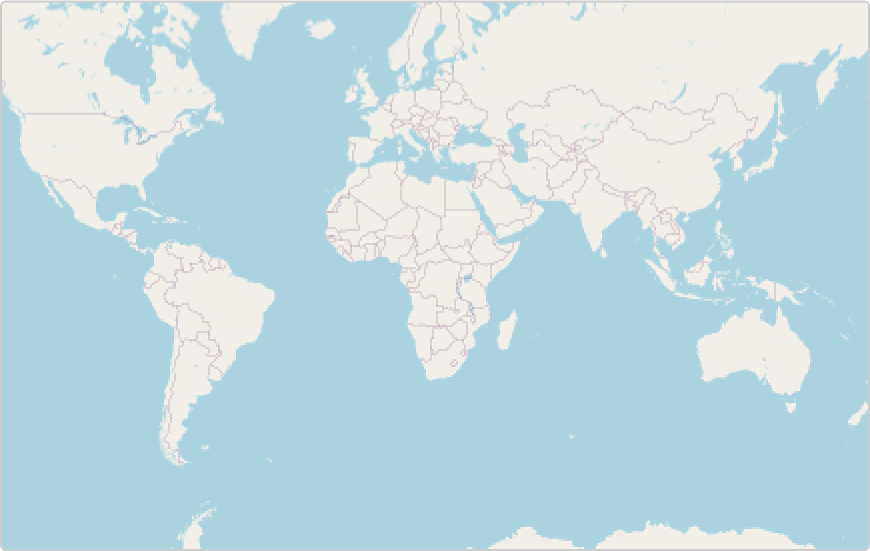

9. សូមដាក់កាលបរិច្ឆេទ Enter a date

yyyy-mm-dd

---

10. ខេត្ត Province

- ◯ មណ្ឌលគិរី Mondulkiri
- ◯ កំពង់ស្ពឺ Kampong Speu

11. ក្រុមអ្នកចូលរួម Target Group

- ◯ អ្នកចូលព្រៃ Forest Goer (>1km from forest)
- ◯ អ្នករស់នៅព្រៃ Forest Dweller (Inside forest or within 1km of forest)
- ◯ ឧទ្យាននរ: Forest Ranger

12. Mondulkiri Village Name

- 01 ពូត្រុំ Tu Trom
- 02 ទួលល្វា Tuol Lvea
- 03 ដេអេ D.A
- 04 ពូជ្រៃចុងផាង Pu Chrei Choung Phang (baro kong)
- 05 អណ្ដូងក្រឡឹង Andoung Kraloeng
- 06 ពូចារ Pu Char
- 07 ពូញ៉ាវ Pu Nhav
- 08 តាំងលាំង Tang Lang
- 09 ក្រុមអភិរក្សព្រៃ Ranger

13. Kampong Speu OD Name

- Phnom Sruoch
- Kampong Speu

14. Phnom Sruoch OD Village Name

- Banteay Roka
- Banteay Roka_Kirisenchey (M)
- Dak Por_Toap Mreak (M)
- Doung_Kraong Meanchey (M)
- Krang Chek
- Krasang Khpos
- Peam Lvea
- Peam Lvea_Sre Doung (M)
- Prey Kahiech
- Rumduol Thmei
- Trapeang Chey_Prey Toteoung (M)

15. Kampong Speu OD Village Name

- ◯ Anlong Sangkae
- ◯ Choam
- ◯ Kampeh
- ◯ Knong Ay
- ◯ Kriel Pong
- ◯ Ou Anchar (M)
- ◯ Roung Masin
- ◯ Ta Sal

16. បុស្តិ៍ ឬស្ថានីយអភិរក្ស Ranger Station Number

____

17. លេខខ្នងផ្ទះ Household Number

____

18. លេខសំគាល់អ្នកចូលរួម Participant ID

MT0-##-##-## or KT0-##-##-## (Province and Timepoint-Village Number-Household Number-Individual Number)

____

Household Information

19. តើមានចំនួនប៉ុន្មាននាក់នៅក្នុងផ្ទះរបស់នាក់? How many people live in your household?

____

24. តើមានប៉ុន្មាននាក់ដែលចូលព្រៃ (យ៉ាងហោចណាស់ចូលព្រៃម្តងក្នុង១សប្តាហ៍)? Of all the people in the household, including yourself, how many people spend time in the forest (visit forest at least once per week)?

____

ព័ត៏មានប្រជាសាស្រ្ត Demographic Information

25. តើការកំណត់យេនឌ័រអ្នកជាអ្វី? What is your gender?

- ◯ ប្រុស Male
- ◯ ស្រី Female
- ◯ ផ្សេងៗ Other
- ◯ សុំមិនបញ្ចេញ Prefer not to say

26. តើអ្នកអាយុប៉ុន្មាន? What is your age?

____

27. តើអ្នកកំណត់អត្តសញ្ញាណ ជនជាតិអ្វី? What ethnic group do you identify with?

- ◯ ខ្មែរ Khmer
- ◯ ប៊ូនង/ព្នង Bunong/Phnong
- ◯ វៀតណាម Vietnamese
- ◯ គ្រឹង Kroeng
- ◯ ទំពួន Tumpoun
- ◯ ចារាយ Charay
- ◯ ព្រៅ Prouv
- ◯ កាចក់ Kachak
- ◯ កាវែត Kavet
- ◯ ឡាវ Lao
- ◯ លន់ Lun
- ◯ ចាម Cham
- ◯ ផ្សេងទៀតបញ្ជាក់ Other

28. ផ្សេងទៀតបញ្ជាក់ Describe "other"

____

29. តើអ្នកអាចយល់ភាសាខ្មែរដែលខ្ញុំកំពុងនិយាយទេ? Can you understand spoken Khmer?

- ◯ បាទ/ចាស Yes
- ◯ ទេ No

30. តើអ្នកអាចនិយាយខ្មែរបានស្ទាត់ទេ? Can you speak Khmer fluently?

- ◯ បាទ/ចាស Yes
- ◯ ទេ No

31. តើអ្នកអាចអានជាភាសាខ្មែរបានទេ? Can you read in Khmer?

- ◯ បាទ/ចាស Yes
- ◯ ទេ No

32. តើអ្នកអាចសរសេរជាភាសាខ្មែរបានទេ? Can you write in Khmer?

- ◯ បាទ/ចាស Yes
- ◯ ទេ No

33. តើអ្នកអាចយល់ភាសាប៊ូនង/ព្នងនិយាយទេ? Can you understand spoken Bunong?

- បាទ/ចាស Yes
- ទេ No

34. តើអ្នកអាចនិយាយភាសាប៊ូនង/ព្នងបានស្ទាត់ឬទេ? Can you speak Bunong fluently?

- បាទ/ចាស Yes
- ទេ No

35. តើអ្នកអាចអានភាសាប៊ូនង/ព្នងបានឬទេ? Can you read Bunong?

- បាទ/ចាស Yes
- ទេ No

36. តើអ្នកអាចសរសេរភាសាប៊ូនង/ព្នងបានឬទេ? Can you write in Bunong?

- បាទ/ចាស Yes
- ទេ No

37. តើអ្នកជាមេគ្រួសារឬទេ? (អ្នកដែលផ្តល់ប្រាក់ចំណូលចំបងដើម្បីផ្គត់ផ្គង់គ្រួសារ) Are you the head of the household? (the person who provides the main income to support the family)

- បាទ/ចាស Yes
- ទេ No

38. តើអ្នកមានតួនាទីអ្វីខ្លះ ទាក់ទងនឹងមេគ្រួសារ? What is your position in the family relative to the head of household?

- ឪពុក Father
- ម្តាយ Mother
- កូនស្រី Daughter
- កូនប្រុស Son
- ជីដូន/ជីតា Grandparent
- បងប្អូនជីដូនមួយ Cousin
- ប្តី Husband
- ប្រពន្ធ Wife
- ផ្សេងទៀត Other

39. ផ្សេងទៀតបញ្ជាក់ Describe Other

ប្រវត្តចូលព្រៃ Forest Exposure

40. តើសកម្មភាពអ្វីខ្លះខាងក្រោមនេះជាប្រភពចំណូលសម្រាប់អ្នក / គ្រួសារអ្នកក្នុងឆ្នាំកន្លងមក? Which of the following activities has been a source of income for you or your family in the past year?

- ☐ កសិករ Farmer
- ☐ កាប់ឈើ Logging
- ☐ លក់ដូរ/អាជីវករផ្សារ Market trader
- ☐ អ្នកប្រមូលអនុផលព្រៃឈើ Forest collector/forager
- ☐ ឧទ្យាននុរក្ស Ranger
- ☐ អ្នកបើកបរ/ ម៉ូតូឌុប Driver / Motorbike taxi
- ☐ ស៊ីឈ្នួល/កម្មករ Day laborer
- ☐ មេដឹកនាំជនជាតិដើម Indigenous leader
- ☐ សិប្បកម្ម Handicrafts (basket weaving, etc.)
- ☐ ចូលនិវត្តន៍ Retired
- ☐ គ្មានការងារធ្វើ Unemployed
- ☐ ផ្សេងទៀត Other

41. ផ្សេងទៀតបញ្ជាក់ Describe Other

____

42. តើអ្នកមានចំណាយពេលនៅក្នុងព្រៃ ឬទៅធ្វើការនៅក្នុងព្រៃទេ?Do you spend time in the forest (at least 1 day per week)?

- ◯ បាទ/ចាស Yes
- ◯ ទេ No

43. តើអ្នកចំណាយពេល ប៉ុន្មានថ្ងៃដែរ នៅក្នុងព្រៃក្នុងមួយសប្តាហ៍ជាមធ្យម នៅរដូវប្រាំង? How many days do you spend in the forest per week, on average, during the dry season?

____

44. តើអ្នកចំណាយពេល ប៉ុន្មានថ្ងៃដែរ នៅក្នុងព្រៃក្នុងមួយសប្តាហ៍ជាមធ្យម នៅរដូវភ្លៀង? How many days do you spend in the forest per week, on average, during the rainy season?

____

ស្ថានភាពការរស់នៅ Living Structures - Asking about both PRIMARY and SECONDARY living structures

45. តើកន្លែងគេងរបស់អ្នកមានលក្ខណៈ/ទម្រង់យ៉ាងដូចម្តេច (ផ្ទះទីមួយ-ដែលអ្នកប្រើញឹកញាប់)? What does your PRIMARY (most used) sleeping structure look like?

- ◯ បន្ទប់ដែលព័ទ្ធជុំវិញមានជញ្ជាំងនិង ដំបូល/ ពិដាន Enclosed room with walls and a ceiling/roof
- ◯ ពិដាននិងជញ្ជាំងខំណ្ឌបន្ទប់ ២-៣ Ceiling and 2-3 walls
- ◯ មានតែពិដានប៉ុណ្ណោះ Only ceiling
- ◯ ចំហរទាំងអស់ Completely open

46. តើសម្ភារៈនៃដំបូល(រចនាសម្ព័ន្ធគេងធម្មតាដែលប្រើញឹកញាប់)ធ្វើពីអ្វី ? What is the material of the ceiling made of in this PRIMARY sleeping structure?

- ☐ ឬស្សី / ស្មៅ / ស្បូវ Bamboo/grass/thatch
- ☐ សន្លឹកប្លាស្ទិក Plastic sheet
- ☐ ប៉ាតង់ Tarpaulin
- ☐ ឈើ Wood
- ☐ សំង្កសី Tin
- ☐ ក្បឿង Tile
- ☐ ផ្សេងទៀត Other

47. ពិពណ៌នាផ្សេងទៀត Describe Other

____

48. តើអ្នកមានរចនាសម្ព័ន្ធគេងទី២ដែរឬទេ? Do you have a SECONDARY sleeping structure (in the forest, near plantation, or similar)?

- ◯ បាទ/ចាស Yes
- ◯ ទេ No

49. ប្រសិនបើអ្នកមានរចនាសម្ព័ន្ធគេងទី២ តើវាមើលទៅមានលក្ខណៈ/ទម្រង់យ៉ាងដូចម្តេច? If you have a SECONDARY sleeping structure (in the forest, near plantation, or similar), what does this secondary sleeping structure look like?

- ◯ បន្ទប់ដែលព័ទ្ធជុំវិញមានជញ្ជាំងនិង ដំបូល/ ពិដាន Enclosed room with walls and a ceiling/roof
- ◯ ពិដាននិងជញ្ជាំងខំណ្ឌបន្ទប់ ២-៣ Ceiling and 2-3 walls
- ◯ មានតែពិដានប៉ុណ្ណោះ Only ceiling
- ◯ ចំហរទាំងអស់ Completely open

50. តើសម្ភារៈនៃដំបូល(រចនាសម្ព័ន្ធគេងទី២)ធ្វើពីអ្វី ? What is the material of the ceiling made of in this SECONDARY sleeping structure?

- [ ] ឫស្សី / ស្មៅ / ស្បូវ Bamboo/grass/thatch
- [ ] សន្លឹកប្លាស្ទិក Plastic sheet
- [ ] ប៉ាតង់ Tarpaulin
- [ ] ឈើ Wood
- [ ] សំង្កសី Tin
- [ ] ក្បឿង Tile
- [ ] ផ្សេងទៀត Other

51. ពិពណ៌នាផ្សេងទៀត Describe Other

---

52. ជារឿយៗតើអ្នកគេងនៅកន្លែងណាក្នុងរដូវភ្លៀង? Where do you normally sleep during the RAINY season?

- [ ] ព្រៃ (រចនាសម្ព័ន្ធគេងញឹកញាប់) Primary sleeping structure in Forest
- [ ] វាលស្រែសើម (រចនាសម្ព័ន្ធគេងញឹកញាប់) Primary sleeping structure in Wet rice fields
- [ ] ចម្ការ (រចនាសម្ព័ន្ធគេងញឹកញាប់) Primary sleeping structure in Plantations
- [ ] ភូមិ (រចនាសម្ព័ន្ធគេងញឹកញាប់) Primary sleeping structure in Villages
- [ ] ព្រៃ (រចនាសម្ព័ន្ធគេងទី២) Secondary sleeping structure in Forest
- [ ] វាលស្រែសើម (រចនាសម្ព័ន្ធគេងទី២) Secondary sleeping structure in Wet rice fields
- [ ] ចម្ការ (រចនាសម្ព័ន្ធគេងទី២) Secondary sleeping structure in Plantations
- [ ] ភូមិ (រចនាសម្ព័ន្ធគេងទី២) Secondary sleeping structure in Villages
- [ ] ផ្សេងទៀត Other

53. ពិពណ៌នាផ្សេងទៀត Describe Other

---

54. ជារឿយៗតើអ្នកគេងនៅកន្លែងណាក្នុងរដូវប្រាំង? Where do you normally sleep during the DRY season?

- [ ] ព្រៃ (រចនាសម្ព័ន្ធគេងញឹកញាប់) Primary sleeping structure in Forest
- [ ] វាលស្រែសើម (រចនាសម្ព័ន្ធគេងញឹកញាប់) Primary sleeping structure in Wet rice fields
- [ ] ចម្ការ (រចនាសម្ព័ន្ធគេងញឹកញាប់) Primary sleeping structure in Plantations
- [ ] ភូមិ (រចនាសម្ព័ន្ធគេងញឹកញាប់) Primary sleeping structure in Villages
- [ ] ព្រៃ (រចនាសម្ព័ន្ធគេងទី២) Secondary sleeping structure in Forest
- [ ] វាលស្រែសើម (រចនាសម្ព័ន្ធគេងទី២) Secondary sleeping structure in Wet rice fields
- [ ] ចម្ការ (រចនាសម្ព័ន្ធគេងទី២) Secondary sleeping structure in Plantations
- [ ] ភូមិ (រចនាសម្ព័ន្ធគេងទី២) Secondary sleeping structure in Villages
- [ ] ផ្សេងទៀត Other

55. ពិពណ៌នាផ្សេងទៀត Describe Other

---

56. តើរបស់របរអ្វីខ្លះដែលយកតាមនៅកំឡុងពេលនៅផ្ទះ និងកន្លែងធ្វើការពេលអ្នកទៅកន្លែងដែលអ្នកបានរៀបរាប់ខាង លើ? Which items do you carry between home and the worksite when you to go the places you mentioned above?

- [ ] កញ្ចប់ចូលព្រៃ Global Fund Forest Pack
- [ ] មុង Bed nets
- [ ] មុងអង្រឹង Hammock nets
- [ ] កន្ទេល Sleeping mats
- [ ] ភួយ Blankets
- [ ] អាហារ Food
- [ ] ទឹក Water
- [ ] ថ្នាំ Medicine
- [ ] ផ្សេងៗ Other

57. ពិពណ៌នាផ្សេងទៀត Describe Other

---

58. តើនៅផ្ទះ/ប៉ុស្តិ៍ របស់អ្នក (ផ្ទះគោល) មានចំងាយប៉ុន្មានពី ព្រៃ? How close is your main (PRIMARY) house or Ranger Station to the forest?

- ( ) ខ្ញុំរស់នៅក្នុងព្រៃ I live in the forest / Ranger Station in the forest
- ( ) ក្នុងចម្ងាយ ១00 ម៉ែត្រ Within 100m
- ( ) ក្នុងចម្ងាយ ២00 ម៉ែត្រ Within 200m
- ( ) ក្នុងចម្ងាយ ៥00 ម៉ែត្រ Within 500m
- ( ) ក្នុងចម្ងាយ ១ គីឡូម៉ែត្រ Within 1km
- ( ) ក្នុងចម្ងាយ ១ ដល់ ២ គីឡូម៉ែត្រ Within 1-2km
- ( ) ចំងាយជាង ២គីឡូម៉ែត្រ Over 2km

វិធីសាស្រ្តការពារខ្លួនពីមូសខាំ Mosquito Bite Prevention

59. តើអ្នកបានធ្វើអ្វីដើម្បីការពារកុំអោយមូសខាំនៅក្នុងផ្ទះរបស់អ្នកទេ នៅពេលថ្ងៃ? Do you do anything to prevent mosquito bites INSIDE your house during DAYTIME?

- ( ) បាទ/ចាស Yes
- ( ) ទេ No
- ( ) មិនដឹង Don't know

60. តើអ្នកបានធ្វើអ្វីដើម្បីការពារកុំអោយមូសខាំនៅក្នុងផ្ទះរបស់អ្នក នៅពេលថ្ងៃ? What do you do to prevent biting INSIDE your house during DAYTIME?

- ☐ ដាំទឹកឱ្យពុះ Boil water
- ☐ ដេកក្នុងមុង Sleep under mosquito net
- ☐ ប្រើថ្នាំបាញ់ថ្នាំសំលាប់សត្វល្អិត Use insecticide spray
- ☐ ប្រើថ្នាំលាបការពារមុសលើស្បែក Use skin repellent
- ☐ ដុតធូបមុស Burn coil
- ☐ ដុតធូប ឬឈើ Burn incense or wood
- ☐ ប្រើមុងអង្រឹង Use hammock net
- ☐ ប្រើសំណាញ់ដាក់បង្អួច ឬទ្វារ Use window or door screen
- ☐ ប្រើសម្លៀកបំពាក់ដៃវែង Use long sleeve clothing
- ☐ ប្រើកញ្ចប់ចូលព្រៃ Use Global Fund forest pack
- ☐ ខ្ញុំមិនដឹង I don't know
- ☐ ផ្សេងទៀត Other

61. ពិពណ៌នាផ្សេងទៀត Describe Other

____

62. តើអ្នកបានធ្វើអ្វីដើម្បីការពារកុំអោយមូសខាំនៅក្នុងផ្ទះរបស់អ្នកទេ នៅពេលយប់? Do you do anything to prevent mosquito bites INSIDE your house during NIGHT TIME?

- ◯ បាទ/ចាស Yes
- ◯ ទេ No
- ◯ មិនដឹង Don't know

63. តើអ្នកធ្វើអ្វីដើម្បីការពារកុំអោយមូសខាំនៅក្នុងផ្ទះរបស់អ្នក នៅពេលយប់? What do you do to prevent biting INSIDE your house during NIGHT TIME?

- ☐ ដាំទឹកឱ្យពុះ Boil water
- ☐ ដេកក្នុងមុង Sleep under mosquito net
- ☐ ប្រើថ្នាំបាញ់ថ្នាំសំលាប់សត្វល្អិត Use insecticide spray
- ☐ ប្រើថ្នាំលាបការពារមុសលើស្បែក Use skin repellent
- ☐ ដុតធូបមុស Burn coil
- ☐ ដុតធូប ឬឈើ Burn incense or wood
- ☐ ប្រើមុងអង្រឹង Use hammock net
- ☐ ប្រើសំណាញ់ដាក់បង្អួច ឬទ្វារ Use window or door screen
- ☐ ប្រើសម្លៀកបំពាក់ដៃវែង Use long sleeve clothing
- ☐ ប្រើកញ្ចប់ចូលព្រៃ Use Global Fund forest pack
- ☐ ខ្ញុំមិនដឹង I don't know
- ☐ ផ្សេងទៀត Other

64. ពិពណ៌នាផ្សេងទៀត Describe Other

____

65. តើអ្នកបានធ្វើអ្វីដើម្បីការពារកុំអោយមូសខាំនៅក្រៅផ្ទះរបស់អ្នកទេ នៅពេលថ្ងៃ? Do you do anything to prevent mosquito bites OUTSIDE your house during DAYTIME?

- ◯ បាទ/ចាស Yes
- ◯ ទេ No
- ◯ មិនដឹង Don't know

66. តើអ្នកបានធ្វើអ្វីដើម្បីការពារកុំអោយមូសខាំនៅក្រៅផ្ទះរបស់អ្នក នៅពេលថ្ងៃ? What do you do to prevent biting OUTSIDE your house during DAYTIME?

- ☐ ដាំទឹកឱ្យពុះ Boil water
- ☐ ដេកក្នុងមុង Sleep under mosquito net
- ☐ ប្រើថ្នាំបាញ់ថ្នាំសំលាប់សត្វល្អិត Use insecticide spray
- ☐ ប្រើថ្នាំលាបការពារមុសលើស្បែក Use skin repellent
- ☐ ដុតធូបមុស Burn coil
- ☐ ដុតធូប ឬឈើ Burn incense or wood
- ☐ ប្រើមុងអង្រឹង Use hammock net
- ☐ ប្រើសំណាញ់ដាក់បង្អួច ឬទ្វារ Use window or door screen
- ☐ ប្រើសម្លៀកបំពាក់ដៃវែង Use long sleeve clothing
- ☐ ប្រើកញ្ចប់ចូលព្រៃ Use Global Fund forest pack
- ☐ ខ្ញុំមិនដឹង I don't know
- ☐ ផ្សេងទៀត Other

67. ពិពណ៌នាផ្សេងទៀត Describe Other

____

68. តើអ្នកបានធ្វើអ្វីដើម្បីការពារកុំអោយមូសខាំនៅក្រៅផ្ទះរបស់អ្នកទេ នៅពេលយប់? Do you do anything to prevent mosquito bites OUTSIDE your house during NIGHT TIME?

- ◯ បាទ/ចាស Yes
- ◯ ទេ No
- ◯ មិនដឹង Don't know

69. តើអ្នកបានធ្វើអ្វីដើម្បីការពារកុំអោយមូសខាំនៅក្រៅផ្ទះរបស់អ្នក នៅពេលយប់? What do you do to prevent biting OUTSIDE your house during NIGHT TIME?

- ☐ ដាំទឹកឱ្យពុះ Boil water
- ☐ ដេកក្នុងមុង Sleep under mosquito net
- ☐ ប្រើថ្នាំបាញ់ថ្នាំសំលាប់សត្វល្អិត Use insecticide spray
- ☐ ប្រើថ្នាំលាបការពារមុសលើស្បែក Use skin repellent
- ☐ ដុតធូបមុស Burn coil
- ☐ ដុតធូប ឬឈើ Burn incense or wood
- ☐ ប្រើមុងអង្រឹង Use hammock net
- ☐ ប្រើសំណាញ់ដាក់បង្អួច ឬទ្វារ Use window or door screen
- ☐ ប្រើសម្លៀកបំពាក់ដៃវែង Use long sleeve clothing
- ☐ ប្រើកញ្ចប់ចូលព្រៃ Use Global Fund forest pack
- ☐ ខ្ញុំមិនដឹង I don't know
- ☐ ផ្សេងទៀត Other

70. ពិពណ៌នាផ្សេងទៀត Describe Other

____

71. ប្រសិនបើអ្នកប្រើផលិតផលការពារមូសខាំ នៅផ្ទះឬក្រៅផ្ទះ តើនរណាជាអ្នកទិញរបស់នោះ? If you use products to prevent mosquito bites inside or outside of your house, who is responsible for purchasing those products?

- ☐ ខ្ញុំមិនប្រើផលិតផលអ្វីទាំងអស់ក្នុងការការពារមិនអោយមូសខាំ I don't use any products to prevent mosquito bites
- ☐ ខ្លួនឯង Self
- ☐ មេគ្រួសារ Head of household
- ☐ ខ្ញុំមិនដឹង I don't know
- ☐ អ្នកផ្សេងទៀត Other
- ☐ មិនបង្ហាញ N/A

72. ពិពណ៌នាអ្នកផ្សេងទៀត Describe Other

____

73. ប្រសិនបើអ្នកទៅព្រៃ/ស្រែ/ចំការ តើអ្នកធ្វើអ្វីដើម្បីការពារមូសខាំ នៅពេលថ្ងៃ? If you go to the FOREST/work site/rice fields/chamkar, what do you do to prevent mosquito bites during DAY TIME?

- ☐ ដាំទឹកឱ្យពុះ Boil water
- ☐ ដេកក្នុងមុង Sleep under mosquito net
- ☐ ប្រើថ្នាំបាញ់ថ្នាំសំលាប់សត្វល្អិត Use insecticide spray
- ☐ ប្រើថ្នាំលាបការពារមុសលើស្បែក Use skin repellent
- ☐ ដុតធូបមុស Burn coil
- ☐ ដុតធូប ឬឈើ Burn incense or wood
- ☐ ប្រើមុងអង្រឹង Use hammock net
- ☐ ប្រើសំណាញ់ដាក់បង្អួច ឬទ្វារ Use window or door screen
- ☐ ប្រើសម្លៀកបំពាក់ដៃវែង Use long sleeve clothing
- ☐ ប្រើកញ្ចប់ចូលព្រៃ Use Global Fund forest pack
- ☐ ខ្ញុំមិនដឹង I don't know
- ☐ ផ្សេងទៀត Other

74. ពិពណ៌នាផ្សេងទៀត **Describe Other**

---

75. ប្រសិនបើអ្នកទៅព្រៃ/ស្រែ/ចំការ តើអ្នកធ្វើអ្វីដើម្បីការពារមូសខាំ នៅពេលយប់? If you go to the FOREST/work site/rice fields/chamkar, what do you do to prevent mosquito bites during NIGHT TIME?

- ☐ ដាំទឹកឱ្យពុះ Boil water
- ☐ ដេកក្នុងមុង Sleep under mosquito net
- ☐ ប្រើថ្នាំបាញ់ថ្នាំសំលាប់សត្វល្អិត Use insecticide spray
- ☐ ប្រើថ្នាំលាបការពារមុសលើស្បែក Use skin repellent
- ☐ ដុតធូបមុស Burn coil
- ☐ ដុតធូប ឬឈើ Burn incense or wood
- ☐ ប្រើមុងអង្រឹង Use hammock net
- ☐ ប្រើសំណាញ់ដាក់បង្អួច ឬទ្វារ Use window or door screen
- ☐ ប្រើសម្លៀកបំពាក់ដៃវែង Use long sleeve clothing
- ☐ ប្រើកញ្ចប់ចូលព្រៃ Use Global Fund forest pack
- ☐ ខ្ញុំមិនដឹង I don't know
- ☐ ផ្សេងទៀត Other

76. ពិពណ៌នាផ្សេងទៀត **Describe Other**

---

77. ប្រសិនបើអ្នកប្រើផលិតផលការពារមូសខាំខណៈនៅព្រៃ តើនរណាជាអ្នកទិញរបស់របរនោះ? If you use products to prevent mosquito bites in the FOREST, who is responsible for purchasing those products?

- ☐ ខ្ញុំមិនបានប្រើប្រាស់ផលិតផលអ្វីទេក្នុងការការពារមូសខាំនោះទេ I don't use any products to prevent mosquito bites
- ☐ ខ្ញុំជាអ្នកទិញ Self
- ☐ មេគ្រួសារ Head of household
- ☐ អ្នកផ្សេងទៀត Other
- ☐ ខ្ញុំមិនដឹងទេ I don't know
- ☐ មិនអាចប្រាប់បាន N/A

78. ពិពណ៌នាអ្នកផ្សេងទៀត Describe Other

____

ការធ្វើដំណើរ និងជម្ងឺគ្រុនចាញ់ Travel and Malaria

79. តើមានចំងាយប៉ុន្មានពីផ្ទះ(ចំបង)អ្នកទៅកន្លែងលក់ឥវ៉ាន់នោះ? How far do you travel to buy things (km from primary household)?

- ◯ ក្នុងចម្ងាយ ៥00 ម៉ែត្រ Within 500m
- ◯ ក្នុងចម្ងាយ ២ គីឡូម៉ែត្រ Within 2km
- ◯ ក្នុងចម្ងាយ ៥ គីឡូម៉ែត្រ Within 5km
- ◯ លើសពី ៥ គីឡូម៉ែត្រ More than 5km

80. តើអ្នកត្រូវបានគេធ្វើរោគវិនិច្ឆ័យថាមានជំងឺគ្រុនចាញ់ប៉ុន្មានដងហើយ (គិតតែចំនួន វិជ្ជមាន)? How many times have you been diagnosed with malaria in your life?

- ◯ មិនដែលទាល់តែសោះ Never
- ◯ ម្តង Once
- ◯ ២ ទៅ ៥ ដង 2-5 times
- ◯ ៦ ទៅ ១០ ដង 6-10 times
- ◯ លើសពី ១០ ដង More than 10 times
- ◯ មិនដឹង I don't know

81. តើនៅចុងក្រោយពេលណា ដែលអ្នកត្រូវបានគេធ្វើរោគវិនិច្ឆ័យថាមានជំងឺគ្រុនចាញ់? When was the last time you were diagnosed with malaria?

- ◯ មិនដែលទាល់តែសោះ Never
- ◯ ក្នុងសប្តាហ៍មុន Last week
- ◯ កាលពីខែមុន Last month
- ◯ កាលពី ៣ ខែមុន Last 3 months
- ◯ កាលពី ៦ ខែមុន Last 6 months
- ◯ កាលពីឆ្នាំមុន Last year
- ◯ ច្រើនជាង ១ ឆ្នាំ More than one year

82. ប្រសិនបើ ឬពេលដែលអ្នកឈឺ [គ្រុនចាញ់] តើអ្នកទៅរកការធ្វើតេស្ត និងព្យាបាលដែរឬទេ? When you feel sick, do you seek testing and treatment?

- ◯ បាទ/ចាស Yes
- ◯ ទេ No

83. ប្រសិនបើ បាទ/ចាស តើអ្នកបានទៅទីណាដើម្បីធ្វើការធ្វើតេស្ត និងព្យាបាល? If yes, where do you seek testing and treatment?

- ☐ អ្នកស្ម័គ្រចិត្តគ្រុនចាញ់ភូមិ (VMW/MMW) Village Malaria Worker
- ☐ ប៉ុស្តិ៍សុខភាព Health Post
- ☐ មណ្ឌលសុខភាព Health Centre
- ☐ មន្ទីរពេទ្យបង្អែក Referral Hospital
- ☐ អ្នកព្យាបាលតាមបែបបុរាណ Traditional healer
- ☐ ផ្សេងទៀត Other

84. ពិពណ៌នាអ្នកផ្សេងទៀត Describe Other

____

85. ប្រសិនបើ ទេ តើហេតុអ្វីបានជាអ្នកមិនទៅទទួលការធ្វើតេស្ត និងព្យាបាល? If no, why do you not seek testing and treatment?

____

86. តើអ្នកបានទទួលថ្នាំលេបមុនពេលទៅព្រៃដើម្បីការពារគ្រុនចាញ់ ពីក្រសួង/ជ្ញាតិបាលដែរឬទេ? Have you received prophylactic medicine from the government to prevent malaria?

- ◯ បាទ/ចាស Yes
- ◯ ទេ No
- ◯ មិនដឹង I don't know

87. ប្រសិនបើ បាទ/ចាស តើអ្នកបានទទួលថ្នាំនេះចុងក្រោយនៅពេលណា? If yes, when did you most recently receive this medicine?

- ◯ ក្នុងសប្តាហ៍មុន Last Week
- ◯ កាលពីខែមុន Last Month
- ◯ កាលពី ៣ ខែមុន Last 3 Months
- ◯ កាលពី ៦ ខែមុន Last 6 Months
- ◯ កាលពីឆ្នាំមុន Last Year
- ◯ ច្រើនជាង ១ ឆ្នាំ More than one year ago

88. Have you spent time in the forest in the last month (within last 30 days)?

- ◯ Yes
- ◯ No
- ◯ I don't know
