# Supplementary material for: Identifying malaria risks amongst forest going populations in Mondulkiri province and Kampong Speu province, Cambodia: a large cross-sectional survey
Source: Malar J. 2025 Feb 22;24:59. doi: 10.1186/s12936-025-05290-0 (PMC11847376; doi:10.1186/s12936-025-05290-0)
Supplement: Supplementary file 3 — Supplementary Material 3 [file 12936_2025_5290_MOESM3_ESM.pdf]

05_CrossSectional_INDIVIDUAL_T1

1. តើបុគ្គលនេះជាអ្នកថ្មីសម្រាប់ការសិក្សាវគ្គនេះឬ? Is this individual a NEW ENROLMENT to the study?

- ◯ បាទ/ចាស ជាអ្នកថ្មី Yes (this person was NOT previously enroled)
- ◯ ទេ! គាត់ជាអ្នកចាស់ (គាត់បានចូលរួមតាំងពីវគ្គមុន) No (this person was in the previous collections)

ការប្រមូលសំណាកឈាម និងការធ្វើតេស្តគ្រុនចាញ់ដោយតេស្តរហ័សBlood Collection and Rapid Test

2. តើអ្នកបានយកសំណាកឈាមពីបុគ្គលនេះដែរឬទេ? Were blood spots collected from this participant?

- ◯ បាទ/ចាស Yes
- ◯ ទេ No

3. ប្រសិនបើបានយក តើបានយកសំណាកឈាមចំនួនប៉ុន្មានចំនុច? If blood spots were collected, how many ?

____

4. តើបុគ្គលនេះបានធ្វើតេស្តរហ័សរកជម្ងឺគ្រុនចាញ់ដែរឬទេ? Was a malaria rapid test given to this participant?

- ◯ បាទ/ចាស Yes
- ◯ ទេ No

5. ប្រសិនបើបានធ្វើតេស្តរហ័ស តើលទ្ធផលតេស្តយ៉ាងដូចម្តេចដែរ? If a rapid test was given, what was the result?

- ◯ អវិជ្ជមាន Negative
- ◯ វិជ្ជមាន Positive
- ◯ មិនអាចសន្និដ្ឋានបាន Inconclusive

ពត៌មានការប្រមូលទិន្នន័យ Data Collection Information

6. លេខសំគាល់ក្រុម Supervisor Number

____

7. ឈ្មោះអ្នកស្រាវជ្រាវ Interviewer name

____

8. សូមកត់ត្រាទីតាំងបច្ចុប្បន្ន Record your current location

latitude (x.y °)

longitude (x.y °)

altitude (m)

accuracy (m)

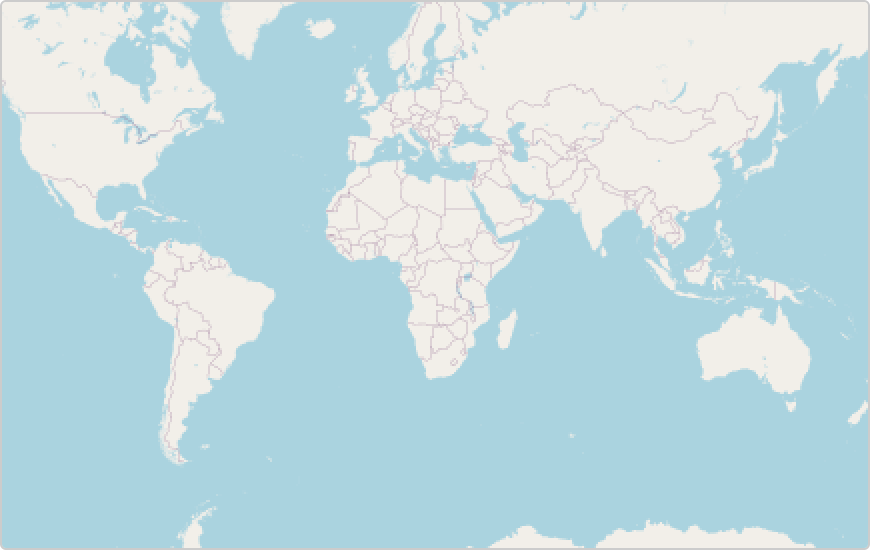

9. សូមដាក់កាលបរិច្ឆេទ Enter a date

yyyy-mm-dd

10. ខេត្ត Province

- ◯ មណ្ឌលគីរី Mondulkiri
- ◯ កំពង់ស្ពឺ Kampong Speu

11. ក្រុមអ្នកចូលរួម Target Group

- ◯ អ្នកចូលព្រៃ Forest Goer (>1km from forest)
- ◯ អ្នករស់នៅភូមិក្នុងព្រៃ Forest Dweller (Inside forest or within 1km of forest)
- ◯ ឧទ្យាននរ: Forest Ranger

12. Mondulkiri Village Name

- 01 ពូត្រុំ Tu Trom
- 02 ទួលល្វា Tuol Lvea
- 03 ដេអេ D.A
- 04 ពូជ្រៃចុងផាង Pu Chrei Choung Phang (baro kong)
- 05 អណ្ដូងក្រឡឹង Andoung Kraloeng
- 06 ពូចារ Pu Char
- 07 ពូញ៉ាវ Pu Nhav
- 08 តាំងលាំង Tang Lang
- 09 ក្រុមអភិរក្សព្រៃ Ranger

13. Kampong Speu OD Name

- Phnom Sruoch
- Kampong Speu

14. Phnom Sruoch OD Village Name

- 01 ក្រាំងចេក Kraing Chek
- 02 រំដួលថ្មី Rumduol Thmei
- 03 ពាមល្វា Peam Lvea
- 04 បន្ទាយរការគិរីសែនជ័យ Banteay Roka_Kirisenchey (M)
- 05 ត្រពាំងជ័យព្រៃទទឹង/ ត្រពាំងកឋិន Trapeang Chey_Prey Toteoung/Trapaing Kathen
- 06 ក្រសាំងខ្ពស់ Krasang Khpos
- 07 ដកពោទំរម្រាក់ Dak Por_Toap Mreak (M)
- 08 ពាមល្វា ស្រែដូង Peam Lvea_Sre Doung (M)
- 09 ព្រៃ កាហៀច Prey Kahiech
- 10 បន្ទាយរការ Banteay Roka
- 11 ដូង ក្រាំងមានជ័យ Doung_Kraong Meanchey (M)

15. Kampong Speu OD Village Name

- 01 ខ្នងអាយ Knong Ay
- 02 សាមគ្គី Samaki
- 03 ពស់វែក Pous Vek
- 04 កំប៉េះ Kampeh
- 05 អន្លង់សង្កែ Anlong Sangkae
- 06 សូរិយា Soriya
- 07 គ្រៀលពង Kriel Pong
- 08 ជាំ Choam

16. បុស្ដិ៍ ឬស្ថានីយអនុរក្ស Ranger Station Number

17. លេខខ្នងផ្ទះ Household Number

18. លេខសំគាល់អ្នកចូលរួម Participant ID

MT1-##-###-## or KT1-##-###-## (Province and Timepoint-Village Number-Household Number-Individual Number). Ex: MT1-01-001-01

ស្ថានភាពការរស់នៅ Living Structures

19. តើមានរចនាសម្ព័ន្ធ(ផ្ទះ ខ្ទម ឬផ្ទះចំការ ។ល។) ផ្សេងៗគ្នាចំនួនប៉ុន្មាន (រួមបញ្ចូលតែផ្ទះ ឬខ្ទម ឬបន្ទប់ចំអិនអាហារ ឬចង្ក្រានបាយ ។ល។) ដែលសមាជិកគ្រួសារចំណាយពេលសម្រាប់គេង ចម្អិនអាហារ។ល។) How many different structures are part of your household (only include structures people spend time in for sleeping, cooking, etc.)

20. សូមពិពណ៌នាពីរចនាសម្ព័ន្ធ (ផ្ទះ ខ្ទម ឬផ្ទះចំការ) ដែលអ្នកប្រើប្រាស់ញឹកញាប់ (អាចបានដល់៥) Please describe the most used structures (up to 5)

Column

រចនាសម្ព័ន្ធទី១ Structure 1

- មានតែពិដានប៉ុណ្ណោះ Only ceiling
- បន្ទប់ដែលព័ទ្ធជុំវិញមានជញ្ជាំងនិង ដំបូល/ ពិដាន Enclosed room with walls and a ceiling/roof
- ពិដាននិងជញ្ជាំងខំណ្ឌបន្ទប់ ២-៣ Ceiling and 2-3 walls
- ចំហរទាំងអស់ Completely open

រចនាសម្ព័ន្ធទី២ Structure 2

- មានតែពិដានប៉ុណ្ណោះ Only ceiling
- បន្ទប់ដែលព័ទ្ធជុំវិញមានជញ្ជាំងនិង ដំបូល/ ពិដាន Enclosed room with walls and a ceiling/roof
- ពិដាននិងជញ្ជាំងខំណ្ឌបន្ទប់ ២-៣ Ceiling and 2-3 walls
- ចំហរទាំងអស់ Completely open

រចនាសម្ព័ន្ធទី៣ Structure 3

- មានតែពិដានប៉ុណ្ណោះ Only ceiling
- បន្ទប់ដែលព័ទ្ធជុំវិញមានជញ្ជាំងនិង ដំបូល/ ពិដាន Enclosed room with walls and a ceiling/roof
- ពិដាននិងជញ្ជាំងខំណ្ឌបន្ទប់ ២-៣ Ceiling and 2-3 walls
- ចំហរទាំងអស់ Completely open

រចនាសម្ព័ន្ធទី៤ Structure 4

- មានតែពិដានប៉ុណ្ណោះ Only ceiling
- បន្ទប់ដែលព័ទ្ធជុំវិញមានជញ្ជាំងនិង ដំបូល/ ពិដាន Enclosed room with walls and a ceiling/roof
- ពិដាននិងជញ្ជាំងខំណ្ឌបន្ទប់ ២-៣ Ceiling and 2-3 walls
- ចំហរទាំងអស់ Completely open

រចនាសម្ព័ន្ធទី៥ Structure 5

- មានតែពិដានប៉ុណ្ណោះ Only ceiling
- បន្ទប់ដែលព័ទ្ធជុំវិញមានជញ្ជាំងនិង ដំបូល/ ពិដាន Enclosed room with walls and a ceiling/roof
- ពិដាននិងជញ្ជាំងខំណ្ឌបន្ទប់ ២-៣ Ceiling and 2-3 walls
- ចំហរទាំងអស់ Completely open

ការធ្វើរោគវិនិច្ឆ័យ និងការព្យាបាលជម្ងឺគ្រុនចាញ់ Malaria Diagnosis and Treatment

21. តើអ្នកត្រូវបានគេធ្វើរោគវិនិច្ឆ័យថាមានជំងឺគ្រុនចាញ់ក្នុងរយៈពេល 60 ថ្ងៃចុងក្រោយនេះទេ (ឬចាប់តាំងពីអ្នកបានជួបជាមួយបុគ្គលិកសិក្សា(ក្រុមយើង)ចុងក្រោយដែរឬទេ? Have you been diagnosed with malaria in the past 60 days (or since you last met with study staff)?

- បាទ/ចាស (វិជ្ជមាន) Yes
- ទេ (អវិជ្ជមាន) No

22. ប្រសិនបើ បាទ/ចាស តើអ្នកបានទទួលការធ្វើរោគវិនិច្ឆ័យជម្ងឺគ្រុនចាញ់នេះនៅឯណា? If yes, where did you receive this diagnosis?

- [ ] អ្នកស្ម័គ្រចិត្ត ព្យាបាលជំងឺគ្រុនចាញ់ភូមិ (VMW/MMW) Village Malaria Worker
- [ ] ប៉ុស្តិ៍សុខភាព Health Post
- [ ] មណ្ឌលសុខភាព Health Centre
- [ ] មន្ទីរពេទ្យបង្អែក Referral Hospital
- [ ] អ្នកព្យាបាលតាមបែបបុរាណ Traditional healer
- [ ] ផ្សេងទៀត Other

23. ពិពណ៌នាផ្សេងទៀត Describe other

____

24. តើអ្នកបានទទួលការព្យាបាលជម្ងឺគ្រុនចាញ់ដែរឬទេ ក្នុងរយៈពេល 60 ថ្ងៃចុងក្រោយនេះទេ (ឬចាប់តាំងពីអ្នកបានជួបជាមួយបុគ្គលិកសិក្សា(ក្រុមយើង)ចុងក្រោយដែរឬទេ? Were you treated for malaria in the past 60 days (or since you last met with study staff)?

- ( ) បាទ/ចាស (បានទទួលការព្យាបាល) Yes
- ( ) ទេ (មិនបានទទួលការព្យាបាលទេ) No

25. ប្រសិនបើ បាទ/ចាស តើអ្នកបានទទួលការព្យាបាលជម្ងឺគ្រុនចាញ់នោះនៅឯណា? If yes, where did you receive this treatment?

- [ ] អ្នកស្ម័គ្រចិត្ត ព្យាបាលជំងឺគ្រុនចាញ់ភូមិ (VMW/MMW) Village Malaria Worker
- [ ] ប៉ុស្តិ៍សុខភាព Health Post
- [ ] មណ្ឌលសុខភាព Health Centre
- [ ] មន្ទីរពេទ្យបង្អែក Referral Hospital
- [ ] អ្នកព្យាបាលតាមបែបបុរាណ Traditional healer
- [ ] ផ្សេងទៀត Other

26. ពិពណ៌នាផ្សេងទៀត Describe other

____

27. តើអ្នកមានគ្រុនក្តៅ (> 98oF ឬ >37អង្សា) ក្នុងរយៈពេលបីថ្ងៃចុងក្រោយនេះទេ? (ប្រសិនបើបាទ/ចាស ធ្វើតេស្តរហ័ស RDTគ្រុនចាញ់) Have you had a fever (>98oF) in the last three days? (IF YES, ADMINISTER RDT)

- ( ) បាទ/ចាស Yes
- ( ) ទេ No
- ( ) ខ្ញុំមិនដឹងទេ I don't know

28. ក្នុងរយៈពេល 60 ថ្ងៃចុងក្រោយនេះ ឬចាប់តាំងពីអ្នកបានឃើញក្រុមការងារយើងចុងក្រោយ តើអ្នកបានទទួលថ្នាំការពារ (បន្ទះភើក) ដែរឬទេ? In the last 60 days, or since you last saw the study team, did you receive a SPATIAL REPELLENT?

- ( ) បាទ/ចាស បានទទួល Yes
- ( ) ទេ (មិនបានទទួលទេ) No
- ( ) ខ្ញុំមិនដឹងទេ I don't know

ថ្នាំការពារតាមលំហអាកម្ម (បន្ទះភើក) Spatial Repellent

29. បើបាន តើអ្នកបានទទួលបន្ទះភើកចំនួនប៉ុន្មានសន្លឹក? (ចំនួនសន្លឹក) If yes, how many did you receive? (# of sheets)

____

30. តើអ្នកបានប្រើថ្នាំការពារតាមលំហអាកម្ម(បន្ទះភើក) របស់កញ្ចប់ព្រៃប៉ែនេះក្នុងរយៈពេល 60 ថ្ងៃចុងក្រោយដែរឬទេ (ឬចាប់តាំងពីអ្នកបានជួបជាមួយពួកយើងចុងក្រោយ)? បើបាទ តើប្រើញឹកញាប់ប៉ុណ្ណា? Have you used the forest pack spatial repellent in the last 60 days (or since you last met with study staff)? If yes, how often?

- ◯ ទេ មិនបានប្រើវាទេ No, I have not used it
- ◯ បាទ/ចាស បានប្រើជារៀងរាល់ថ្ងៃ Yes, every day
- ◯ បាទ/ចាស បានប្រើវាចន្លោះពី ២ទៅ៦ ថ្ងៃក្នុង ១សប្តាហ៍ (ស្ទើរតែរៀងរាល់ថ្ងៃ) Yes, 2-6 days per week (most days)
- ◯ បាទ/ចាស បានប្រើវាម្តងក្នុង ១សប្តាហ៍ Yes, once per week
- ◯ បាទ/ចាស បានប្រើវាម្តងក្នុង ២សប្តាហ៍ Yes, once every two weeks
- ◯ បាទ/ចាស បានប្រើវាម្តងក្នុង ១ខែ Yes, once a month

31. តើអ្នកបានប្រើថ្នាំការពារតាមលំហអាកម្ម (បន្ទះភើក) កាលពីម្សិលមិញ ឬយប់មិញដែរឬទេ? Did you use the spatial repellent yesterday or last night?

- ◯ បាទ/ចាស បានប្រើ Yes
- ◯ ទេ មិនបានប្រើទេ No

32. ប្រសិនបើអ្នកបានប្រើថ្នាំការពារតាមលំហអាកម្ម (បន្ទះភើក) តើអ្នកបានប្រើវានៅទីណា? If you used the spatial repellent, WHERE did you use it?

- ☐ ក្នុងផ្ទះរបស់ខ្ញុំ In my house
- ☐ នៅខាងក្រៅ ក្បែរផ្ទះខ្ញុំ Outside, nearby my house
- ☐ នៅក្នុងរចនាសម្ព័ន្ធ (ផ្ទះឬខ្ទម)ក្នុងព្រៃ របស់ខ្ញុំ In my living structure in the forest
- ☐ ផ្សេងៗ Other

33. ពិពណ៌នាផ្សេងៗ Describe other

____

34. ប្រសិនបើអ្នកប្រើប្រាស់ថ្នាំការពារតាមលំហអាកម្ម (បន្ទះភើក)។ តើហេតុអ្វីបានជាអ្នកប្រើប្រាស់វា? If you used the spatial repellent, WHY did you use it?

- ☐ ដើម្បីបណ្តេញមូស To keep mosquitoes away
- ☐ ខ្ញុំត្រូវបានលើកទឹកចិត្តឱ្យប្រើវា I was encouraged to use it
- ☐ វាងាយស្រួលប្រើ It is easy to use
- ☐ ខ្ញុំបានកត់សម្គាល់ឃើញមូសតិចនៅពេលខ្ញុំប្រើវា I noticed fewer mosquitoes when I use it
- ☐ សម្លៀកបំពាក់ផ្សេងទៀតរបស់ខ្ញុំគឺកខ្វក់ My other clothing was dirty
- ☐ មិនដឹង I don't know
- ☐ ផ្សេងៗ Other

35. ពិពណ៌នាផ្សេងៗ Describe other

36. ប្រសិនបើអ្នកមិនបានប្រើប្រាស់ថ្នាំការពារតាមលំហអាកាស (បន្ទះភើក)។ តើហេតុអ្វីបានជាអ្នកមិនប្រើប្រាស់វា? If you did NOT use the spatial repellent, why not?

- ☐ ខ្ញុំគិតថាវាគ្មានប្រសិទ្ធភាពទេ I don't think it works
- ☐ វាមានភាពមិនសូវងាយស្រួលសម្រាប់ខ្ញុំទេ It's not convenient for me
- ☐ ខ្ញុំបារម្ភថាវាអាចធ្វើអោយពុល ឬគ្រោះថ្នាក់បាន I'm worried it's toxic or dangerous
- ☐ ខ្ញុំមិនចូលចិត្តវាទេ I don't like how it feels
- ☐ ខ្ញុំមិនចូលចិត្តក្លិនវាទេ I don't like the way it smells
- ☐ ខ្ញុំមិនចូលចិត្តរូបរាង ឬទម្រង់វា I don't like how it looks
- ☐ ខ្ញុំមិនដឹង I don't know
- ☐ ផ្សេងៗ Other

37. ពិពណ៌នាផ្សេងៗ Describe other

38. តើអ្នកទំនងនឹងណែនាំថ្នាំការពារទំហំអាកាសនេះដល់សមាជិកគ្រួសារ ឬមិត្តភ័ក្តិយ៉ាងណាដែរ? How likely would you be to recommend the spatial repellent to a family member or friend?

- ◯ ច្បាស់ជាមិនណែនាំតែម្តង Strongly would NOT recommend
- ◯ នឹងមិនណែនាំ Would NOT recommend
- ◯ គ្មានយោបល់ No opinon
- ◯ នឹងណែនាំ Would recommend
- ◯ ច្បាស់ជានឹងណែនាំខ្លាំងតែម្តង Strongly would recommend

39. តើអ្នកមានអារម្មណ៍ថាថ្នាំការពារលំហអាកាសមានប្រយោជន៍ប៉ុណ្ណាក្នុងជីវិតប្រចាំថ្ងៃរបស់អ្នក? How useful do you feel the spatial repellent is in your day-to-day life?

- ◯ គ្មានប្រយោជន៍ Useless
- ◯ មិនមានប្រយោជន៍ខ្លាំងណាស់ណាទេ Not very useful
- ◯ គ្មានយោបល់ No opinion
- ◯ មានប្រយោជន៍ខ្លះ Somewhat useful
- ◯ មានប្រយោជន៍ណាស់ Very useful

40. តើអ្នកធ្លាប់មានបទពិសោធន៍ណាមួយខាងក្រោមក្នុងរយៈពេល 60 ថ្ងៃចុងក្រោយនេះទេ (ឬចាប់តាំងពីអ្នកបានជួបជាមួយបុគ្គលិកសិក្សាយើងចុងក្រោយ) ដែលអ្នកជឿថាបណ្តាលមកពីការប្រើថ្នាំលំហអកម្មនេះ? Did you experience any of the following in the last 60 days (or since you last met with study staff), which you believe was due to the spatial repellent?

- [ ] គ្មានរោគសញ្ញាអ្វីទាំងអស់ None
- [ ] កន្ទួលលើទ្រូង Rash on chest
- [ ] កន្ទួលលើខ្នង Rash on back
- [ ] កន្ទួលលើជើង Rash on legs
- [ ] កន្ទួលលើដៃ Rash on arms
- [ ] រលាកភ្នែក Irritation or burning in eyes
- [ ] រលាកច្រមុះ Irritation or burning in nose
- [ ] រលាកមាត់ Irritation or burning in mouth
- [ ] ដង្ហើមខ្លី Shortness of breath
- [ ] ពិបាកដកដង្ហើម Difficulty breathing
- [ ] ក្អក Coughing
- [ ] ក្អក (ក្អកមានស្លេស) Productive (with mucous) cough
- [ ] ប្រតិកម្មអាឡែស៊ី Allergic reaction
- [ ] ឈឺក្បាល Headache
- [ ] ផ្សេងៗ Other

40b. ពិពណ៌នាផ្សេងៗ Describe other

____

41. តើរោគសញ្ញាខាងលើមានរយៈពេលប៉ុន្មានថ្ងៃ? ប្រសិនបើមានរោគសញ្ញាច្រើនជាងមួយ សូមជ្រើសរើសមួយណាដែលមានរយៈពេលវែងបំផុត។ How many days did the symptom(s) last? If more than one symptom, choose longest duration.

____

42. តើអ្នកបានធ្វើអ្វីមួយដើម្បីដោះស្រាយរោគសញ្ញានោះទេ? Did you take any action to resolve the symptoms?

- ( ) បាទ/ចាស បាន Yes
- ( ) ទេ No

43. តើកាលមានរោគសញ្ញាខាងលើ អ្នកតម្រូវអោយមានការព្យាបាលដែរឬទេ? Was treatment of the symptoms required?

- ( ) បាទ/ចាស Yes
- ( ) ទេ No

44. តើអ្នកបានទៅគ្រឹះស្ថានសុខាភិបាលណាមួយដោយសាររោគសញ្ញានឹងដែរឬទេ? Did you visit a health facility due to the symptoms?

- ( ) បាទ/ចាស បាន Yes
- ( ) ទេ No

45. ប្រសិនបើបាទ/ចាសបានទៅ តើអ្នកបានទៅទីកន្លែងណា? If yes, where did you seek treatment?

- [ ] អ្នកស្ម័គ្រចិត្ត ព្យាបាលជំងឺគ្រុនចាញ់ភូមិ (VMW/MMW) Village Malaria Worker
- [ ] ប៉ុស្តិ៍សុខភាព Health Post
- [ ] មណ្ឌលសុខភាព Health Centre
- [ ] មន្ទីរពេទ្យបង្អែក Referral Hospital
- [ ] អ្នកព្យាបាលតាមបែបបុរាណ Traditional healer
- [ ] ផ្សេងទៀត Other

46. ពិពណ៌នាផ្សេងៗ Describe other

____

47. តើអ្នកបានសំរាកព្យាបាលប៉ុន្មានយប់នៅគ្រឹះស្ថានសុខាភិបាលនោះដែរឬទេ? Did you have to stay overnight at the health facility?

- ( ) បាទ/ចាស Yes
- ( ) ទេ No

48. តើអ្នកបានចំណាយលើការព្យាបាលនោះដែរឬទេ? Did you have to pay for your treatment?

- ( ) បាទ/ចាស បានចំណាយ Yes
- ( ) ទេ មិនបានចំណាយទេ No

49. ប្រសិនបើបានចំណាយ តើអ្នកបានចំណាយប្រាក់អស់ប៉ុន្មាន? If yes, how much did you pay (Riel)?

____

50. តើរោគសញ្ញានោះបានជាសះស្បើយដែរឬទេ? Have your symptoms resolved?

- ( ) បាទ/ចាស Yes
- ( ) ទេ No

51. ក្នុងរយៈពេល 60 ថ្ងៃចុងក្រោយ ឬចាប់តាំងពីអ្នកបានឃើញក្រុមការងារយើងចុងក្រោយ តើអ្នកបានទទួលថ្នាំលាបលើស្បែកការពារមូសដែរឬទេ? In the last 60 days, or since you last saw the study team, did you receive a TOPICAL REPELLENT?

- ( ) បាទ/ចាស បានទទួលថ្នាំលាបលើស្បែក Yes
- ( ) ទេ មិនបានទទួលថ្នាំលាបលើស្បែកទេ No
- ( ) មិនដឹង I don't know

ថ្នាំលាបលើស្បែកការពារមូស Topical Repellent

52. ប្រសិនបើបានទទួលថ្នាំលាបលើស្បែក តើបានទទួលប៉ុន្មានដប? If yes, how many topical repellent units did you receive? (1 bottle = 1 unit)

____

53. តើអ្នកបានប្រើថ្នាំលាបលើស្បែករបស់កញ្ចប់ចូលព្រៃប៉ៃនេះក្នុងរយៈពេល 60 ថ្ងៃចុងក្រោយដែរឬទេ (ឬចាប់តាំងពីអ្នកបានជួបជាមួយពួកយើងចុងក្រោយ)? បើបាទ តើប្រើញឹកញាប់ប៉ុណ្ណា? Have you used the forest pack topical repellent in the last 60 days (or since you last met with study staff)? If yes, how often?

- ◯ ទេ មិនបានប្រើវាទេ No, I have not used it
- ◯ បាទ/ចាស បានប្រើជារៀងរាល់ថ្ងៃ Yes, every day
- ◯ បាទ/ចាស បានប្រើវាចន្លោះពី ២ទៅ៦ ថ្ងៃក្នុង ១សប្តាហ៍ (ស្ទើរតែរៀងរាល់ថ្ងៃ) Yes, 2-6 days per week (most days)
- ◯ បាទ/ចាស បានប្រើវាម្តងក្នុង ១សប្តាហ៍ Yes, once per week
- ◯ បាទ/ចាស បានប្រើវាម្តងក្នុង ២សប្តាហ៍ Yes, once every two weeks
- ◯ បាទ/ចាស បានប្រើវាម្តងក្នុង ១ខែ Yes, once a month

54. តើអ្នកបានប្រើថ្នាំលាបលើស្បែកកាលពីម្សិលមិញ ឬយប់មិញដែរឬទេ? Did you use the topical repellent yesterday or last night?

- ◯ បាទ/ចាស បានប្រើ Yes
- ◯ ទេ មិនបានប្រើទេ No

55. ប្រសិនបើអ្នកបានប្រើថ្នាំលាបលើស្បែក តើអ្នកបានប្រើវានៅទីណា? If you used the topical repellent, WHERE did you use it?

- ☐ ក្នុងផ្ទះរបស់ខ្ញុំ In my house
- ☐ នៅខាងក្រៅ ក្បែរផ្ទះខ្ញុំ Outside, nearby my house
- ☐ នៅក្នុងរចនាសម្ព័ន្ធ (ផ្ទះឬខ្ទម)ក្នុងព្រៃ របស់ខ្ញុំ In my living structure in the forest
- ☐ ផ្សេងៗ Other

56. ពិពណ៌នាផ្សេងៗ Describe other

___

57. ប្រសិនបើអ្នកបានប្រើថ្នាំលាបលើស្បែក មូលហេតុអ្វីបានជាអ្នកប្រើវា? If you used the topical repellent, WHY did you use it?

- ☐ ដើម្បីបណ្តេញមូស To keep mosquitoes away
- ☐ ខ្ញុំត្រូវបានលើកទឹកចិត្តឱ្យប្រើវា I was encouraged to use it
- ☐ វាងាយស្រួលប្រើ It is easy to use
- ☐ ខ្ញុំបានកត់សម្គាល់ឃើញមូសតិចនៅពេលខ្ញុំប្រើវា I noticed fewer mosquitoes when I use it
- ☐ សម្លៀកបំពាក់ផ្សេងទៀតរបស់ខ្ញុំគឺកខ្វក់ My other clothing was dirty
- ☐ មិនដឹង I don't know
- ☐ ផ្សេងៗ Other

58. ពិពណ៌នាផ្សេងៗ Describe other

___

59. ប្រសិនបើអ្នកមិនបានប្រើប្រាស់ថ្នាំលាបស្បែក។ តើហេតុអ្វីបានជាអ្នកមិនប្រើប្រាស់វា? If you did NOT use the topical repellent, why not?

- [ ] ខ្ញុំគិតថាវាគ្មានប្រសិទ្ធភាពទេ I don't think it works
- [ ] វាមានភាពមិនសូវងាយស្រួលសម្រាប់ខ្ញុំទេ It's not convenient for me
- [ ] ខ្ញុំបារម្ភថាវាអាចធ្វើអោយពុល ឬគ្រោះថ្នាក់បាន I'm worried it's toxic or dangerous
- [ ] ខ្ញុំមិនចូលចិត្តវាទេ I don't like how it feels
- [ ] ខ្ញុំមិនចូលចិត្តក្លិនវាទេ I don't like the way it smells
- [ ] ខ្ញុំមិនចូលចិត្តរូបរាង ឬទម្រង់វា I don't like how it looks
- [ ] ខ្ញុំមិនដឹង I don't know
- [ ] ផ្សេងៗ Other

60. ពិពណ៌នាផ្សេងៗ Describe other

____

61. តើអ្នកទំនងនឹងណែនាំថ្នាំលាបស្បែកនេះដល់សមាជិកគ្រួសារ ឬមិត្តភ័ក្តិយ៉ាងណាដែរ? How likely would you be to recommend the topical repellent to a family member or friend?

- ○ ច្បាស់ជាមិនណែនាំតែម្តង Strongly would NOT recommend
- ○ នឹងមិនណែនាំ Would NOT recommend
- ○ គ្មានយោបល់ No opinon
- ○ នឹងណែនាំ Would recommend
- ○ ច្បាស់ជានឹងណែនាំខ្លាំងតែម្តង Strongly would recommend

62. តើអ្នកមានអារម្មណ៍ថាថ្នាំលាបស្បែកមានប្រយោជន៍ប៉ុណ្ណាក្នុងជីវិតប្រចាំថ្ងៃរបស់អ្នក? How useful do you feel the topical repellent is in your day-to-day life?

- ○ គ្មានប្រយោជន៍ Useless
- ○ មិនមានប្រយោជន៍ខ្លាំងណាស់ណាទេ Not very useful
- ○ គ្មានយោបល់ No opinion
- ○ មានប្រយោជន៍ខ្លះ Somewhat useful
- ○ មានប្រយោជន៍ណាស់ Very useful

63. តើអ្នកធ្លាប់មានបទពិសោធន៍ណាមួយខាងក្រោមក្នុងរយៈពេល 60 ថ្ងៃចុងក្រោយនេះទេ (ឬចាប់តាំងពីអ្នកបានជួបជាមួយបុគ្គលិកសិក្សាយើងចុងក្រោយ) ដែលអ្នកជឿថាបណ្តាលមកពីការថ្នាំលាបលើស្បែកនេះ? Did you experience any of the following in the last 60 days (or since you last met with study staff), which you believe was due to the topical repellent?

- ☐ គ្មានរោគសញ្ញាអ្វីទាំងអស់ None
- ☐ កន្ទួលលើទ្រូង Rash on chest
- ☐ កន្ទួលលើខ្នង Rash on back
- ☐ កន្ទួលលើជើង Rash on legs
- ☐ កន្ទួលលើដៃ Rash on arms
- ☐ រលាកភ្នែក Irritation or burning in eyes
- ☐ រលាកច្រមុះ Irritation or burning in nose
- ☐ រលាកមាត់ Irritation or burning in mouth
- ☐ ដង្ហើមខ្លី Shortness of breath
- ☐ ពិបាកដកដង្ហើម Difficulty breathing
- ☐ ក្អក Coughing
- ☐ ក្អក (ក្អកមានស្លេស) Productive (with mucous) cough
- ☐ ប្រតិកម្មអាឡែស៊ី Allergic reaction
- ☐ ឈឺក្បាល Headache
- ☐ ផ្សេងៗ Other

63b. ពិពណ៌នាផ្សេងៗ Describe other

____

64. តើរោគសញ្ញាខាងលើមានរយៈពេលប៉ុន្មានថ្ងៃ? ប្រសិនបើមានរោគសញ្ញាច្រើនជាងមួយ សូមជ្រើសរើសមួយណាដែលមានរយៈពេលវែងបំផុត។ How many days did the symptom(s) last? If more than one symptom, choose longest duration.

____

65. តើអ្នកបានធ្វើអ្វីមួយដើម្បីដោះស្រាយរោគសញ្ញានោះទេ? Did you take any action to resolve the symptoms?

- ○ បាទ/ចាស បាន Yes
- ○ ទេ No

66. តើរោគសញ្ញាខាងលើនោះ តម្រូវអោយអ្នកធ្វើការព្យាបាលដែរឬទេ? Was treatment of the symptoms required?

- ○ បាទ/ចាស Yes
- ○ ទេ No

67. តើអ្នកបានទៅគ្រឹះស្ថានសុខាភិបាលណាមួយព្យាបាលដោយសាររោគសញ្ញានោះដែរឬទេ? Did you visit a health facility due to the symptoms?

- ○ បាទ/ចាស បាន Yes
- ○ ទេ No

68. ប្រសិនបើបាទ/ចាសបានទៅ តើអ្នកបានទៅទីកន្លែងណា? If yes, where did you seek treatment?

- ☐ អ្នកស្ម័គ្រចិត្ត ព្យាបាលជំងឺគ្រុនចាញ់ភូមិ (VMW/MMW) Village Malaria Worker
- ☐ ប៉ុស្តិ៍សុខភាព Health Post
- ☐ មណ្ឌលសុខភាព Health Centre
- ☐ មន្ទីរពេទ្យបង្អែក Referral Hospital
- ☐ អ្នកព្យាបាលតាមបែបបុរាណ Traditional healer
- ☐ ផ្សេងទៀត Other

69. ពិពណ៌នាផ្សេងៗ Describe other

____

70. តើអ្នកបានសំរាកព្យាបាល នៅគ្រឹះស្ថានសុខាភិបាលនោះដែលឬទេ? Did you have to stay overnight at the health facility?

- ○ បាទ/ចាស Yes
- ○ ទេ No

71. តើអ្នកបានចំណាយលើការព្យាបាលនោះដែលឬទេ? Did you have to pay for your treatment?

- ○ បាទ/ចាស បានចំណាយ Yes
- ○ ទេ មិនបានចំណាយទេ No

72. ប្រសិនបើបានចំណាយ តើអ្នកបានចំណាយប្រាក់អស់ប៉ុន្មានរៀល? If yes, how much did you pay (Riel)?

____

73. តើរោគសញ្ញានោះបានជាសះស្បើយដែលឬទេ? Have your symptoms resolved?

- ○ បាទ/ចាស Yes
- ○ ទេ No

74. ក្នុងរយៈពេល 60 ថ្ងៃចុងក្រោយនេះ ឬចាប់តាំងពីអ្នកបានឃើញក្រុមការងារយើងចុងក្រោយ តើអ្នកបានជ្រលក់ឬបាញ់ខោអាវអ្នកដោយថ្នាំការពារសត្វល្អិតដែលឬទេ? In the last 60 days, or since you last saw the study team, did you have any of your CLOTHING TREATED WITH INSECTICIDE??

- ○ បាទ/ចាស បាន Yes
- ○ ទេ មិនបានទេ No
- ○ មិនដឹង I don't know

ការជ្រលក់ ឬបាញ់ថ្នាំសត្វល្អិតឬមូសលើសំលៀកបំពាក់ Treated Clothing

75. បើបាន តើអ្នកបានជ្រលក់ចំនួនប៉ុន្មានអាវ ឬខោ? If yes, how many articles of your clothing was treated? (1 shirt = 1 article; 1 pants = 1 article)

____

76. តើអ្នកបានស្លៀក ឬពាក់ខោអាវជ្រលក់ថ្នាំរបស់កញ្ចប់ព្រៃ ប៉ែ នេះក្នុងរយៈពេល 60 ថ្ងៃចុងក្រោយដែរឬទេ (ឬចាប់តាំងពីអ្នកបានជួបជាមួយពួកយើងចុងក្រោយ)? បើបាទ តើប្រើញឹកញាប់ប៉ុណ្ណា? Have you worn the forest pack treated clothing in the last 60 days (or since you last met with study staff)? If yes, how often?

- ◯ ទេ មិនបានប្រើវាទេ No, I have not used it
- ◯ បាទ/ចាស បានប្រើជារៀងរាល់ថ្ងៃ Yes, every day
- ◯ បាទ/ចាស បានប្រើវាចន្លោះពី ២ទៅ៦ ថ្ងៃក្នុង ១សប្តាហ៍ (ស្ទើរតែរៀងរាល់ថ្ងៃ) Yes, 2-6 days per week (most days)
- ◯ បាទ/ចាស បានប្រើវាម្តងក្នុង ១សប្តាហ៍ Yes, once per week
- ◯ បាទ/ចាស បានប្រើវាម្តងក្នុង ២សប្តាហ៍ Yes, once every two weeks
- ◯ បាទ/ចាស បានប្រើវាម្តងក្នុង ១ខែ Yes, once a month

77. តើអ្នកបានស្លៀក ឬពាក់ខោអាវជ្រលក់ថ្នាំកាលពីម្សិលមិញ ឬយប់មិញដែរឬទេ? Did you wear any treated clothing yesterday or last night?

- ◯ បាទ/ចាស បានប្រើ Yes
- ◯ ទេ មិនបានប្រើទេ No

78. ប្រសិនបើអ្នកបានស្លៀក ឬពាក់ខោអាវជ្រលក់ថ្នាំ តើអ្នកបានប្រើវានៅទីណា? If you wore the treated clothing, WHERE did you wear it?

- ☐ ក្នុងផ្ទះរបស់ខ្ញុំ In my house
- ☐ នៅខាងក្រៅ ក្បែរផ្ទះខ្ញុំ Outside, nearby my house
- ☐ នៅក្នុងរចនាសម្ព័ន្ធ (ផ្ទះឬខ្ទម)ក្នុងព្រៃ របស់ខ្ញុំ In my living structure in the forest
- ☐ ផ្សេងៗ Other

79. ពិពណ៌នាផ្សេងៗ Describe other

____

80. ប្រសិនបើអ្នកបានស្លៀក ឬពាក់ខោអាវជ្រលក់ថ្នាំ។ តើហេតុអ្វីបានជាអ្នកស្លៀក ឬពាក់វា? If you wore the treated clothing, WHY did you wear it?

- ☐ ដើម្បីបណ្តេញមូស To keep mosquitoes away
- ☐ ខ្ញុំត្រូវបានលើកទឹកចិត្តឱ្យប្រើវា I was encouraged to use it
- ☐ វាងាយស្រួលប្រើ It is easy to use
- ☐ ខ្ញុំបានកត់សម្គាល់ឃើញមូសតិចនៅពេលខ្ញុំប្រើវា I noticed fewer mosquitoes when I use it
- ☐ សម្លៀកបំពាក់ផ្សេងទៀតរបស់ខ្ញុំគឺកខ្វក់ My other clothing was dirty
- ☐ មិនដឹង I don't know
- ☐ ផ្សេងៗ Other

81. ពិពណ៌នាផ្សេងៗ Describe other

____

82. ប្រសិនបើអ្នកមិនបានស្លៀក ឬពាក់ខោអាវជ្រលក់ថ្នាំ។ តើហេតុអ្វីបានជាអ្នកមិនស្លៀក ឬពាក់វា? If you did NOT wear the treated clothing, why not?

- ☐ ខ្ញុំគិតថាវាគ្មានប្រសិទ្ធភាពទេ I don't think it works
- ☐ វាមានភាពមិនសូវងាយស្រួលសម្រាប់ខ្ញុំទេ It's not convenient for me
- ☐ ខ្ញុំបារម្ភថាវាអាចធ្វើអោយពុល ឬគ្រោះថ្នាក់បាន I'm worried it's toxic or dangerous
- ☐ ខ្ញុំមិនចូលចិត្តវាទេ I don't like how it feels
- ☐ ខ្ញុំមិនចូលចិត្តក្លិនវាទេ I don't like the way it smells
- ☐ ខ្ញុំមិនចូលចិត្តរូបរាង ឬទម្រង់វា I don't like how it looks
- ☐ ខ្ញុំមិនដឹង I don't know
- ☐ ផ្សេងៗ Other

83. ពិពណ៌នាផ្សេងៗ Describe other

____

84. តើអ្នកទំនងនឹងណែនាំខោអាវជ្រលក់ថ្នាំនេះដល់សមាជិកគ្រួសារ ឬមិត្តភ័ក្តិយ៉ាងណាដែរ? How likely would you be to recommend the treated clothing to a family member or friend?

- ◯ ច្បាស់ជាមិនណែនាំតែម្តង Strongly would NOT recommend
- ◯ នឹងមិនណែនាំ Would NOT recommend
- ◯ គ្មានយោបល់ No opinon
- ◯ នឹងណែនាំ Would recommend
- ◯ ច្បាស់ជានឹងណែនាំខ្លាំងតែម្តង Strongly would recommend

85. តើអ្នកមានអារម្មណ៍ថាខោអាវជ្រលក់ថ្នាំមានប្រយោជន៍ប៉ុណ្ណាក្នុងជីវិតប្រចាំថ្ងៃរបស់អ្នក? How useful do you feel the treated clothing is in your day-to-day life?

- ◯ គ្មានប្រយោជន៍ Useless
- ◯ មិនមានប្រយោជន៍ខ្លាំងណាស់ណាទេ Not very useful
- ◯ គ្មានយោបល់ No opinion
- ◯ មានប្រយោជន៍ខ្លះ Somewhat useful
- ◯ មានប្រយោជន៍ណាស់ Very useful

86. តើអ្នកធ្លាប់មានបទពិសោធន៍ណាមួយខាងក្រោមក្នុងរយៈពេល 60 ថ្ងៃចុងក្រោយនេះទេ (ឬចាប់តាំងពីអ្នកបានជួបជាមួយបុគ្គលិកសិក្សាយើងចុងក្រោយ) ដែលអ្នកជឿថាបណ្តាលមកពីខោអាវជ្រលក់ថ្នាំនេះ? Did you experience any of the following in the last 60 days (or since you last met with study staff), which you believe was due to the treated clothing?

- [ ] គ្មានរោគសញ្ញាអ្វីទាំងអស់ None
- [ ] កន្ទួលលើទ្រូង Rash on chest
- [ ] កន្ទួលលើខ្នង Rash on back
- [ ] កន្ទួលលើជើង Rash on legs
- [ ] កន្ទួលលើដៃ Rash on arms
- [ ] រលាកភ្នែក Irritation or burning in eyes
- [ ] រលាកច្រមុះ Irritation or burning in nose
- [ ] រលាកមាត់ Irritation or burning in mouth
- [ ] ដង្ហើមខ្លី Shortness of breath
- [ ] ពិបាកដកដង្ហើម Difficulty breathing
- [ ] ក្អក Coughing
- [ ] ក្អក (ក្អកមានស្លេស) Productive (with mucous) cough
- [ ] ប្រតិកម្មអាឡែស៊ី Allergic reaction
- [ ] ឈឺក្បាល Headache
- [ ] ផ្សេងៗ Other

86b. ពិពណ៌នាផ្សេងៗ Describe other

____

87. តើរោគសញ្ញាខាងលើមានរយៈពេលប៉ុន្មានថ្ងៃ? ប្រសិនបើមានរោគសញ្ញាច្រើនជាងមួយ សូមជ្រើសរើសមួយណាដែលមានរយៈពេលវែងបំផុត។ How many days did the symptom(s) last? If more than one symptom, choose longest duration.

____

88. តើអ្នកបានធ្វើអ្វីមួយដើម្បីដោះស្រាយរោគសញ្ញានោះទេ? Did you take any action to resolve the symptoms?

- ( ) បាទ/ចាស បាន Yes
- ( ) ទេ No

89. តើអ្នកបានទៅរកសេវាគ្រឹះស្ថានសុខាភិបាលណាមួយនៅពេលអ្នកមានរោគសញ្ញានោះដែរឬទេ? Did you visit a health facility due to the symptoms?

- ( ) បាទ/ចាស បាន Yes
- ( ) ទេ No

90. តើរោគសញ្ញាខាងលើនោះ តម្រូវអោយអ្នកធ្វើការព្យាបាលដែរឬទេ? Was treatment of the symptoms required?

- ( ) បាទ/ចាស Yes
- ( ) ទេ No

91. ប្រសិនបើបាទ/ចាសបានទៅ តើអ្នកបានទៅទីកន្លែងណា? If yes, where did you seek treatment?

- [ ] អ្នកស្ម័គ្រចិត្ត ព្យាបាលជំងឺគ្រុនចាញ់ភូមិ (VMW/MMW) Village Malaria Worker
- [ ] ប៉ុស្តិ៍សុខភាព Health Post
- [ ] មណ្ឌលសុខភាព Health Centre
- [ ] មន្ទីរពេទ្យបង្អែក Referral Hospital
- [ ] អ្នកព្យាបាលតាមបែបបុរាណ Traditional healer
- [ ] ផ្សេងទៀត Other

92. ពិពណ៌នាផ្សេងៗ Describe other

____

93. តើអ្នកបានគេងយប់នៅគ្រឹះស្ថានសុខាភិបាលនោះដែរឬទេ? Did you have to stay overnight at the health facility?

- ( ) បាទ/ចាស Yes
- ( ) ទេ No

94. តើអ្នកបានចំណាយលើការព្យាបាលនោះដែរឬទេ? Did you have to pay for your treatment?

- ( ) បាទ/ចាស បានចំណាយ Yes
- ( ) ទេ មិនបានចំណាយទេ No

95. ប្រសិនបើបានចំណាយ តើអ្នកបានចំណាយប្រាក់អស់ប៉ុន្មានរៀល? If yes, how much did you pay (Riel)?

____

96. តើរោគសញ្ញានោះបានជាសះស្បើយដែរឬទេ? Have your symptoms resolved?

- ( ) បាទ/ចាស Yes
- ( ) ទេ No

97. តើអ្នកបានបាត់ផលិតផលណាមួយរបស់កញ្ចប់ចូលព្រៃបែរដែរឬទេ ក្នុងអំឡុងពេល 60 ថ្ងៃចុងក្រោយនេះទេ (ឬចាប់តាំងពីអ្នកបានជួបក្រុមសិក្សាចុងក្រោយ)? Did you lose any of the forest pack products over the last 60 days (or since you last met with the study team)?

- [ ] ទេ No
- [ ] បាទ/ចាស បានបាត់ថ្នាំការពារតាមលំហអកាស(បន្ទះភើក) Yes, the spatial repellent
- [ ] បាទ/ចាស បានបាត់ថ្នាំលាបលើស្បែក Yes, the topical repellent
- [ ] បាទ/ចាស បានបាត់សំលៀកបំពាក់ជ្រលក់ឬបាញ់ថ្នាំ Yes, the treated clothing

ការការពារពីការមូសខាំ Mosquito Bite Prevention

98. ក្នុងរយះពេល 60 ថ្ងៃចុងក្រោយដែរឬទេ (ឬចាប់តាំងពីអ្នកបានជួបជាមួយពួកយើងចុងក្រោយ)? នៅក្នុងផ្ទះរបស់អ្នក តើអ្នកបានធ្វើអ្វីខ្លះក្រៅពីការប្រើប្រាស់កញ្ចប់ចូលព្រៃប៉ៃដើម្បីការពារកុំអោយមូសខាំដែរឬទេ? In the last 60 days (or since you last met with the study team), did you do anything to prevent mosquito bites INSIDE your house that wasn't part of the forest pack??

- ◯ បាទ/ចាស បាន Yes
- ◯ ទេ មិនបានទេ No

99. ប្រសិនបើបាទ/ចាស បាន។ តើអ្នកបានធ្វើអ្វីខ្លះ(នៅក្នុងផ្ទះ)? If yes, what did you use?

- ☐ ដាំទឹកឱ្យពុះ Boil water
- ☐ ដេកក្នុងមុង Sleep under mosquito net
- ☐ ប្រើថ្នាំបាញ់ថ្នាំសំលាប់សត្វល្អិត Use insecticide spray
- ☐ ប្រើថ្នាំលាបការពារមុសលើស្បែក Use skin repellent
- ☐ ដុតធូបមុស Burn coil
- ☐ ដុតធូប ឬឈើ Burn incense or wood
- ☐ ប្រើមុងអង្រឹង Use hammock net
- ☐ ប្រើសំណាញ់ដាក់បង្អួច ឬទ្វារ Use window or door screen
- ☐ ប្រើសម្លៀកបំពាក់ដៃវែង Use long sleeve clothing
- ☐ ប្រើកញ្ចប់ចូលព្រៃ Use Global Fund forest pack
- ☐ ខ្ញុំមិនដឹង I don't know
- ☐ ផ្សេងទៀត Other

100. ពិពណ៌នាផ្សេងៗ Describe other

___

101. ក្នុងរយះពេល 60 ថ្ងៃចុងក្រោយដែរឬទេ (ឬចាប់តាំងពីអ្នកបានជួបជាមួយពួកយើងចុងក្រោយ)? នៅក្រៅផ្ទះរបស់អ្នក តើអ្នកបានធ្វើអ្វីខ្លះក្រៅពីការប្រើប្រាស់កញ្ចប់ចូលព្រៃប៉ៃដើម្បីការពារកុំអោយមូសខាំដែរឬទេ? In the last 60 days (or since you last met with the study team), did you do anything to prevent mosquito bites OUTSIDE your house that wasn't part of the forest pack??

- ◯ បាទ/ចាស បាន Yes
- ◯ ទេ មិនបានទេ No

102. ប្រសិនបើបាទ/ចាស បាន។ តើអ្នកបានធ្វើអ្វីខ្លះ(នៅក្រៅផ្ទះ)? If yes, what did you use?

- ☐ ដាំទឹកឱ្យពុះ Boil water
- ☐ ដេកក្នុងមុង Sleep under mosquito net
- ☐ ប្រើថ្នាំបាញ់ថ្នាំសំលាប់សត្វល្អិត Use insecticide spray
- ☐ ប្រើថ្នាំលាបការពារមុសលើស្បែក Use skin repellent
- ☐ ដុតធូបមុស Burn coil
- ☐ ដុតធូប ឬឈើ Burn incense or wood
- ☐ ប្រើមុងអង្រឹង Use hammock net
- ☐ ប្រើសំណាញ់ដាក់បង្អួច ឬទ្វារ Use window or door screen
- ☐ ប្រើសម្លៀកបំពាក់ដៃវែង Use long sleeve clothing
- ☐ ប្រើកញ្ចប់ចូលព្រៃ Use Global Fund forest pack
- ☐ ខ្ញុំមិនដឹង I don't know
- ☐ ផ្សេងទៀត Other

103. ពិពណ៌នាផ្សេងៗ Describe other

____

ការធ្វើដំណើរ និងពេលវេលាដែលនៅក្នុងព្រៃ Travel and Time Spent in Forest

104. តើអ្នកបានចំណាយពេលនៅក្នុងព្រៃក្នុងរយៈពេល 14 ថ្ងៃចុងក្រោយដែរឬទេ? បើបាន តើប៉ុន្មានដង? Have you spent time in the forest in the last 14 days? If yes, how much time?

- ◯ ទេ មិនបានទេ No
- ◯ បាទ/ចាស បានរាល់ថ្ងៃ Yes, every day
- ◯ បាទ/ចាស បាន ប៉ុន្តែមិនរាល់ថ្ងៃទេ Yes, but not every day

105. ប្រសិនបើ បាទ/ចាស បាន ប៉ុន្តែមិនរាល់ថ្ងៃទេ ថាតើប្រហែលជាចំណាយពេលក្នុងព្រៃប៉ុន្មានថ្ងៃដែរក្នុងពេល 14 ថ្ងៃចុងក្រោយនេះ? If yes, but not every day, estimate the number of days spent in the forest in the last 14 days

____

106. បើបានទៅ។ ហេតុអ្វីបានជាអ្នកទៅព្រៃ? If yes, why did you go to the forest?

- ☐ ខ្ញុំរស់នៅក្នុងព្រៃ I live in the forest
- ☐ ធ្វើការ Working
- ☐ ធ្វើដំណើរទៅភូមិមួយទៀត Travelling to another village
- ☐ ផ្សេងៗ Other

107. ពិពណ៌នាផ្សេងៗ Describe other

____

108. បើបានទៅ។ តើអ្នកទៅជាមួយនរណា? If yes, who did you go to the forest with?

- ☐ មនុស្សក្នុងភូមិរបស់អ្នក People from your village
- ☐ មនុស្សពីភូមិផ្សេង People from other villages

109. If people from other villages, which ones (Mondulkiri - list all)?

___

110. If people from other villages, which ones (Kampong Speu - list all)?

___

111. តើអ្នកបានទៅលេងភូមិផ្សេងទៀតទេក្នុងរយៈពេល 30 ថ្ងៃចុងក្រោយនេះ? Have you visited any other villages in the last 30 days?

- ◯ បាទ/ចាស បាន Yes
- ◯ ទេ មិនបានទេ No

112. ប្រសិនបាទ/ចាសបានទៅជាមួយ ថាតើគាត់មកពីភូមិមួយណា (សម្រាប់ខេត្តមណ្ឌលគីរី) If yes, which villages? (Mondulkiri - Enter all)

___

113. ប្រសិនបាទ/ចាសបានទៅជាមួយ ថាតើគាត់មកពីភូមិមួយណា (សម្រាប់ខេត្តកំពង់ស្ពឺ) If yes, which villages? (Kampong Speu - Enter all)

___
