# Supplementary material for: Identifying malaria risks amongst forest going populations in Mondulkiri province and Kampong Speu province, Cambodia: a large cross-sectional survey
Source: Malar J. 2025 Feb 22;24:59. doi: 10.1186/s12936-025-05290-0 (PMC11847376; doi:10.1186/s12936-025-05290-0)

Table S1. Household amenities

Characteristic*		Total (n=1,303)	Forest Goer (n=711)	Forest Dweller (n=592)
**Primary water source**	Bottled water	30%	26%	34%
	Tube well or bore hole	13%	11%	16%
	Rainwater	9%	10%	8%
	Surface water	18%	22%	12%
	Other	30%	31%	30%
**Primary toilet**	Flush toilet to pit latrine	47%	42%	53%
	Pit latrine with slab	11%	9%	14%
	No toilet / bush / forest	34%	40%	27%
	Other	8%	9%	6%
**Does the household have electricity?**		61%	65%	56%

Table S2. Participant demographics by province (T0 and T1)

Characteristic			n (%)	Mondulkiri	Kampong Speu
Basic demographic data collected on all new participants (T0 and T1)					
**Total individuals**		N (%)	2,935 (100%)	1,510 (51%)	1,425 (49%)
**Province**		Mondulkiri	1,510 (51%)	100%	0%
		Kampong Speu	1,425 (49%)	0%	100%
**Age (years)**		mean (SD)	32.7 (15.5)	32.9 (14.7)	32.5 (16.2)
		<18	517 (18%)	15%	20%
		18-25	540 (18%)	20%	17%
		26-45	1,253 (43%)	45%	40%
		46-65	563 (19%)	19%	20%
		>65	62 (2%)	1%	3%
**Gender**		Male	1,434 (49%)	49%	49%
		Female	1,493 (51%)	51%	51%
		Other/Not specified	4 (<1%)	<1%	0%
Detailed demographic data collected from new participants at T0 only					
**Total individuals**		n	2,111 (100%)	1,104	1,007
**Ethnic group**		Khmer	1,444 (68%)	40%	100%
		Bunong	650 (31%)	59%	0%
		Other	17 (1%)	1%	0%
**Languages (% Yes)**	**Khmer**	Understand spoken	2,100 (99%)	99%	99%
		Speak fluently	2,014 (95%)	93%	98%
		Reading	1,259 (60%)	57%	63%
		Writing	1,210 (57%)	55%	60%
	**Bunong**	Understand spoken	753 (36%)	68%	1%
		Speak fluently	673 (32%)	61%	<1%
		Reading	139 (7%)	13%	0%
		Writing	107 (5%)	10%	0%
**Household position**		Head of household (% total)	879 (42%)	41%	43%
		Spouse of head (husband/wife)	434 (35%)	37%	34%
		Child of head (son/daughter)	668 (54%)	51%	58%
		Parent of head (father/mother)	26 (2%)	2%	2%
		Other	104 (9%)	10%	6%

Table S3. Individual sources of income by province (T0)

Income Sources*	Total (%)	Mondulkiri	Kampong Speu
**Total**	2,111 (100%)	1,104	1,007
**Farmer**	1,687 (47.43%)	870 (52%)	817 (43%)
**Day Labourer**	644 (18.11%)	87 (5%)	326 (17%)
**Forest Collector / Forager**	578 (16.25%)	188 (11%)	390 (21%)
**Other**	200 (5.62%)	95 (6%)	105 (6%)
**Logging**	180 (5.06%)	50 (3%)	130 (7%)
**Market Trader**	149 (4.19%)	75 (4%)	76 (4%)
**Unemployed**	59 (1.66%)	26 (2%)	33 (2%)
**Forest Ranger**	37 (1.04%)	36 (2%)	1 (<1%)
**Driver / Motorbike Taxi**	11 (0.31%)	233 (14%)	10 (1%)
**Retired**	9 (0.25%)	7 (<1%)	2 (<1%)
**Handicrafts (basket weaving, etc.)**	3 (0.08%)	6 (<1%)	2 (<1%)

*Individuals may list more than one source of income

Table S4. Living structure characteristics by province (T0)

Structure characteristics	Total (n=2,111)	Mondulkiri (n=1,104)	Kampong Speu (n=1,007)
Primary living structure			
**Enclosed room with walls and a ceiling/roof**	71%	71%	72%
**Ceiling and 2-3 walls**	28%	28%	28%
**Only ceiling**	<1%	<1%	0%
**Completely open**	<1%	<1%	0%
Secondary living structure			
**Have secondary structure in the forest or farm?**	39%	57%	20%
**Enclosed room with walls and a ceiling/roof**	33%	37%	21%
**Ceiling and 2-3 walls**	5%	5%	3%
**Only ceiling**	44%	35%	74%
**Completely open**	17%	22%	1%

Table S5. Time spent in forest by province (T1 and T2)

Time spent in the forest in last two weeks	Total (n=4,239)	Mondulkiri (n=2,202)	Kampong Speu (n=2,037)
**Did not go to forest**	632 (15%)	314 (14%)	318 (16%)
**Went to forest every day**	2,889 (68%)	1,453 (66%)	1,436 (71%)
**Went to forest but not every day**	716 (17%)	435 (20%)	283 (14%)
**Average number of days***	5.1 (SD 3.2, range 1-14)	5.7 (SD 3.4, range 1-14)	4.0 (SD 2.5, range 1-13)

*For those who went to the forest

Figure S1. P. falciparum and P. vivax qPCR-detected infections in Mondulkiri province. *(Circle = P. falciparum, triangle = P. vivax)*

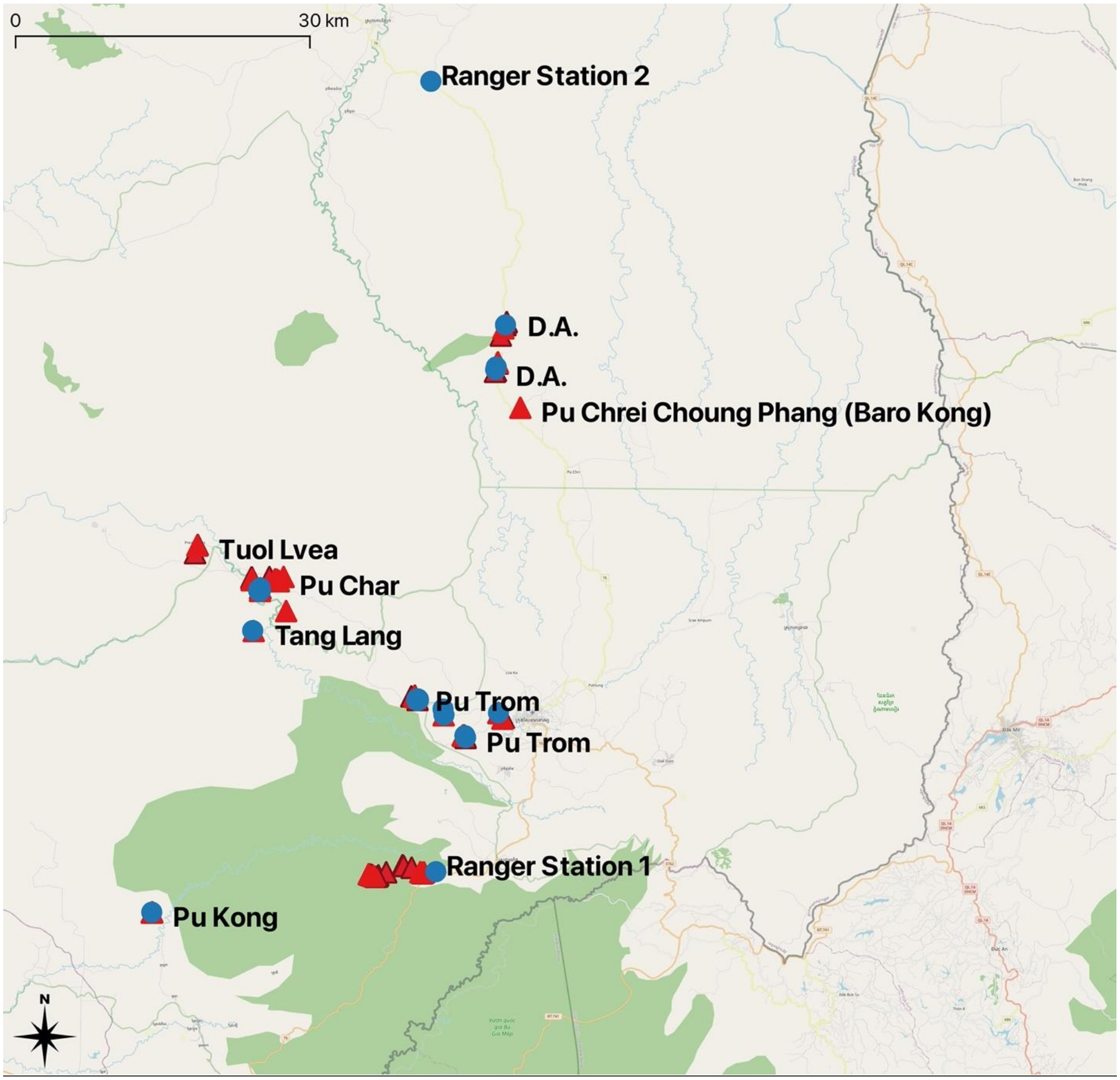

Figure S2. P. falciparum and P. vivax qPCR positive infections in Kampong Speu province.
(Circle = P. falciparum, triangle = P. vivax)

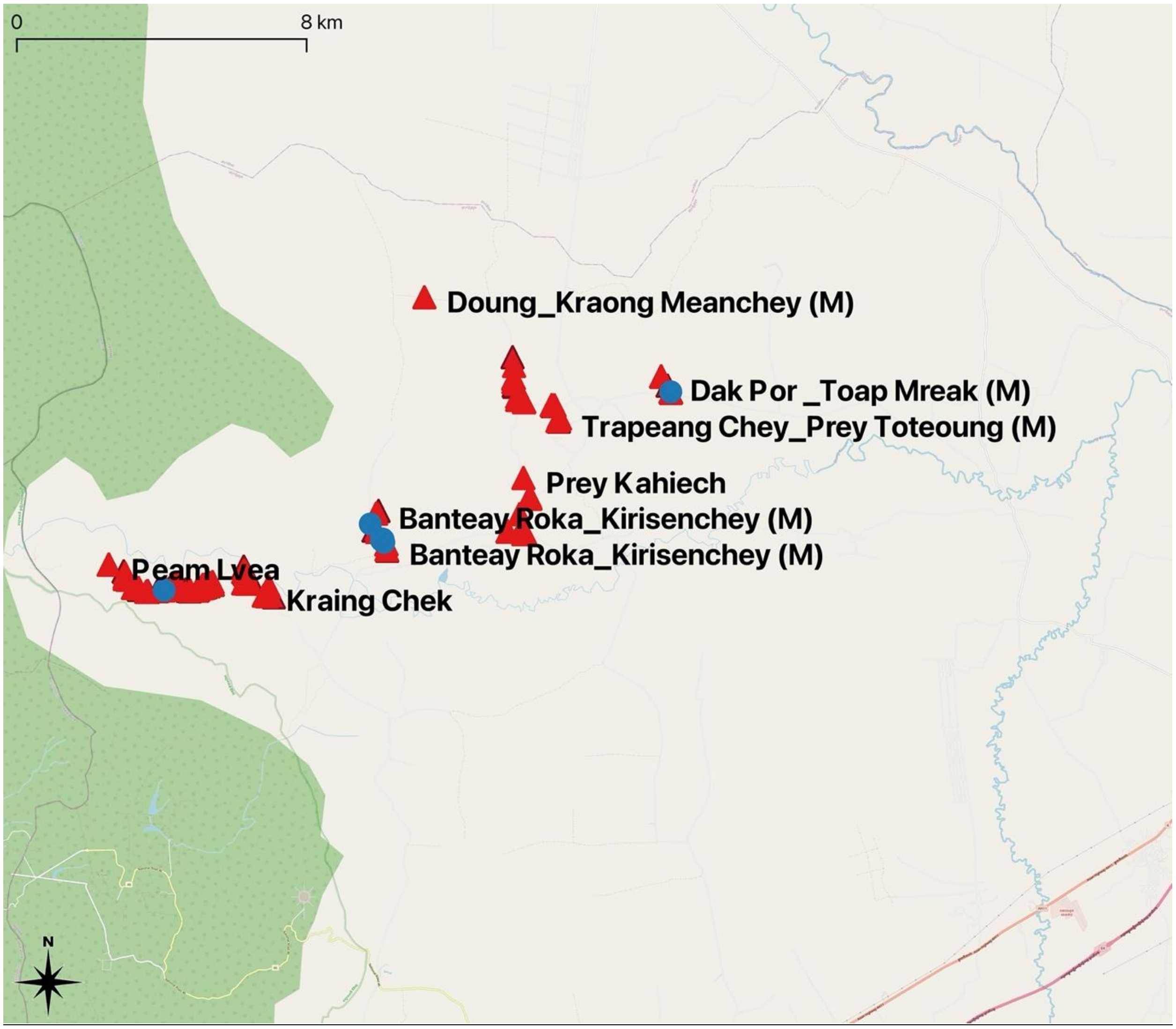

Supplement: Supplementary file 4 — Supplementary Material 4 [file 12936_2025_5290_MOESM4_ESM.pdf]
